# Jitterbug: somatic and germline transposon insertion detection at single-nucleotide resolution

**DOI:** 10.1186/s12864-015-1975-5

**Published:** 2015-10-12

**Authors:** Elizabeth Hénaff, Luís Zapata, Josep M. Casacuberta, Stephan Ossowski

**Affiliations:** Genomic and Epigenomic Variation in Disease Group, Centre for Genomic Regulation (CRG), The Barcelona Institute of Science and Technology, Dr. Aiguader 88, 08003 Barcelona, Spain; Center for Research in Agricultural Genomics, CRAG (CSIC-IRTA-UAB-UB), Barcelona, Spain; Universitat Pompeu Fabra (UPF), Barcelona, Spain; current address: Weill Cornell Medical College, Institute for Computational Biomedicine, 1305 York Avenue, New York, NY 10021 USA

**Keywords:** Transposons, Mobile elements, NGS, Somatic mutation, Cancer, Structural variation, Genomics, Evolution

## Abstract

**Background:**

Transposable elements are major players in genome evolution. Transposon insertion polymorphisms can translate into phenotypic differences in plants and animals and are linked to different diseases including human cancer, making their characterization highly relevant to the study of genome evolution and genetic diseases.

**Results:**

Here we present Jitterbug, a novel tool that identifies transposable element insertion sites at single-nucleotide resolution based on the pairedend mapping and clipped-read signatures produced by NGS alignments. Jitterbug can be easily integrated into existing NGS analysis pipelines, using the standard BAM format produced by frequently applied alignment tools (e.g. bwa, bowtie2), with no need to realign reads to a set of consensus transposon sequences. Jitterbug is highly sensitive and able to recall transposon insertions with a very high specificity, as demonstrated by benchmarks in the human and *Arabidopsis* genomes, and validation using long PacBio reads. In addition, Jitterbug estimates the zygosity of transposon insertions with high accuracy and can also identify somatic insertions.

**Conclusions:**

We demonstrate that Jitterbug can identify mosaic somatic transposon movement using sequenced tumor-normal sample pairs and allows for estimating the cancer cell fraction of clones containing a somatic TE insertion. We suggest that the independent methods we use to evaluate performance are a step towards creating a gold standard dataset for benchmarking structural variant prediction tools.

**Electronic supplementary material:**

The online version of this article (doi:10.1186/s12864-015-1975-5) contains supplementary material, which is available to authorized users.

## Background

Transposable elements (TEs) are mobile genetic elements that account for an important fraction of both plant and animal genomes. Far from being simply selfish elements, TEs contribute extensively to genomes’ function. The complex enzymatic machinery TEs encode, as well as their regulatory elements and even DNA sequence attributes have been repeatedly co-opted by their host genome during evolution [1]. A paradigmatic example are the RAG proteins responsible for the V(D)J recombination system in immunoglobulin, whose DNA-binding and nuclease functions derive from domesticated transposases [2]. Similarly, several transcription factors in both plants and animals derive from TEs [1], as well as promoters [3] and transcription factor binding sites [4, 5]. However, the most obvious impact of TEs is due to their mobility, and the polymorphisms they generate are a rich source of genetic variants that can be selected during evolution. Indeed, transposon-related polymorphisms are at the origin of an important fraction of variability relevant to plant genome evolution both in the wild and for breeding [6, 7], and have strongly affected human evolution [8]. Moreover, at a much shorter timescale, somatic insertions can have an important impact on the phenotype of an individual organism. In plants, somatic mutations induced by transposable element insertions (TEIs) are at the origin of agriculturally relevant traits such as variations in grape color [9] or cluster shape [10]. In humans, the L1 retrotransposon is highly active during neuronal development, and L1 insertions may modify the expression pattern of nearby genes, contributing to neuron diversification [11, 12]. However, TE movement may also lead to disease in humans. For example, increased TE activity in neurons may lead to diseases such as schizophrenia [13] and TEIs have been associated to other human diseases such as hepatocellular carcinoma [14], lung squamous, head and neck, colorectal and endometrial carcinomas [15], as well as to other cancer types [16]. Therefore, the analysis of TE insertion polymorphisms is an important component in studying the evolution of plant and animal genomes and is also highly relevant in the context of elucidating the genetic basis of disease, including cancers. Indeed, tumor development is an evolutionary process in which mutations beneficial to the cancer (e.g. conferring increased proliferation) are selected for. New mutations are acquired over time, and if selected for form a new proliferating sub-clone. Thus, the identification of somatic TEIs in cancer is highly relevant to the study of disease evolution, and remains a challenge as it requires highly sensitive methods able to identify TEIs in a minor fraction of cells (i.e. tumor sub-clones).

The question of identifying the locus of new TE insertions has been addressed in many different ways, including molecular biology techniques (Sequence-Specific Amplification Polymorphism (SSAP), hemi-specific PCR) in specific individuals or plant varieties, but this is not feasible for a large number of elements or samples. Assembly-based approaches have been used comparing BACs [17] or whole genomes [18], which have the advantage of yielding the sequence of the element that is present (or absent) in either genome, and thus enabling sequence comparisons between elements. However these are limited by the amount and quality of assembled genomes available, making this approach less feasible for large numbers of samples, or for highly repetitive genomes. Array-based methods are notoriously blind to the “difficult”, repetitive regions of the genome [19], and the two latter methods preclude the identification of heterozygosity. While mapping of single-end or concordant paired-end sequences can be useful for determining copy number variation (CNV) of genes using depth of coverage, the large copy number of most TE families excludes this approach, as the variations in copy number would be insignificant with respect to the total number of copies. Discordantly mapped paired-end reads have been used to map polymorphic TE sites in human populations (combined with 454 data, [20], Alus) or human cancer lines ([15, 16], LINEs), in the hominid lineage [21] and in plants [22, 23].

The number of studies highlighting the impact of recent TE insertions in evolution and disease supports the importance of including TE-related variant detection among the usual suspects of genome-wide variant studies, such as single nucleotide polymorphisms (SNPs), short indels and CNVs. The proliferation of large datasets of NGS-based paired-end sequencing data provides a goldmine for addressing the genetic basis of trait evolution and disease. To date, there exists a handful of software tools that aim to identify TEIs using paired-end sequencing data, each with their particularities and limitations. For example TEA [16], RetroSeq [24] and VariationHunter [25, 26] all focus on human non-LTR TEs such as Alus, L1 and SVA, and none predict the zygosity of the called insertion. Most available tools (e.g. VariationHunter, TEA) do not accept out-of-the-box BAM alignment files as produced by widely applied alignment tools like bwa, bwa-mem and bowtie1/2. VariationHunter and TEA require alignment of reads against a library of transposon sequences in addition to the genome, which is impractical when the sequencing data is supplied already aligned. Recently, TranspoSeq has been used to identify TEIs in cancer cell lines, and is the only tool to predict the zygosity of the insertions. However, it is designed specifically for, and is limited to, paired tumor-normal datasets in human and requires an LSF cluster environment [15]. TEMP [27] is designed to detect TEIs in pooled samples for population studies, and requires a curated set of TE consensus sequences. Thus the development of a bioinformatics tool to detect TEIs based on mapping signatures of NGS reads, indiscriminately of the type of TE and genome, which operates on standard BAM files, predicts allelic frequency and optionally processes tumor-normal pairs would represent a significant technical advance in the field of structural variant analysis. These characteristics imply relevant applications from the study of plant and animal evolution to human disease, with specific advantages in large scale, storage-heavy projects as the PanCancer Analysis of Whole-Genomes (PCAWG) project (analyzing 2500 cancer tumor-normal pairs), which are limited to a single alignment format due to high storage and computational demands.

Here we present Jitterbug, a tool that identifies novel transposable element insertions in a sequenced sample with respect to a reference genome, based solely on the mapped reads in BAM format and the annotation of TEs in the reference. Jitterbug can be used in any genome for which a reference sequence and TE annotation is available, and detects TEIs of all TE classes. In addition, it predicts the allelic frequency (zygosity) of the insertion as well as optionally compares tumor-normal sample pairs to call somatic insertions even at a low (below 50 %) cancer cell fraction.

## Results and discussion

### An algorithm for identification of transposon insertion sites using paired-end and clipped reads

**Fig. 1:**
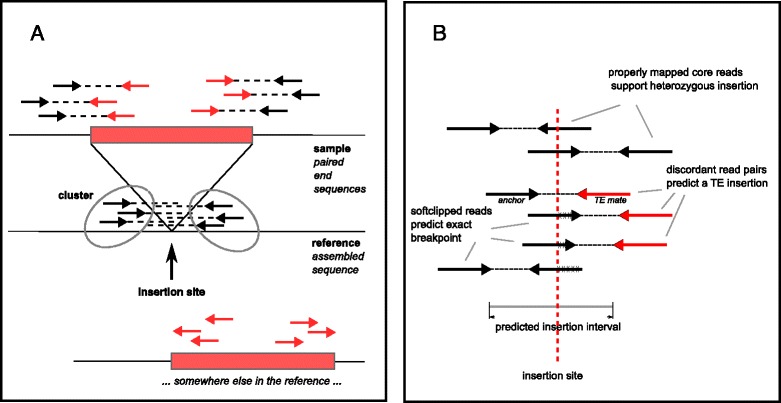
Principal elements of Jitterbug algorithm. **a**) Discordantly mapping read-pairs of which one read maps to an annotated TE predict an insertion event. **b**) Clipped reads are used to narrow down the breakpoint, and properly mapped reads spanning that breakpoint (core reads) indicate the presence of a reference allele. The predicted insertion interval lies between the innermost anchor reads of the forward and reverse clusters

Jitterbug has been designed to identify TE insertions present in samples sequenced with a paired-end approach that are not present in the corresponding reference genome. The algorithm relies on the presence of read pairs that span the TE insertion (TEI) site on either side of the inserted sequence. Such read pairs have one read coming from the sequence near the insertion site and the other from the TE sequence itself. As TEs are usually found in multiple similar copies throughout the genome, the newly inserted TE will likely be similar to another annotated TE in the reference. Therefore such read pairs will map at a discordant distance, with one read (the “anchor”) mapping to a unique genomic location near the insertion site, and the other (the “TE mate”) mapping to a TE similar to the one inserted but found elsewhere in the reference (Fig. 1a). Each of these discordant reads potentially predicts an insertion within an interval (the size of the expected fragment length) downstream of the anchor read (relative to its strand). Sets of overlapping anchor reads are clustered together on either strand, and a pair of forward and reverse clusters which overlap in their prediction interval are considered to predict a putative TE insertion. Mapping software such as bwa [28] will truncate or “soft-clip” reads that consist of two segments mapping to distinct locations, retaining the mapping position of the longer fragment. Reads that overlap the borders of the inserted TE are thus “soft-clipped” (subsequently referred to as “clipped”) and are used to narrow down the prediction interval, the clipped site indicating the exact insertion breakpoint (Fig. 1b). Properly mapped reads that overlap the predicted insertion breakpoint indicate the absence of a TEI, i.e. the reference allele, while the clipped reads indicate the “presence” (non-reference TEI) allele. The ratio of clipped to properly mapped reads at the insertion site represent the allelic frequency (AF) of the insertion, a ratio of 1 indicating homozygosity for the TE insertion, and a ratio around 0.5 a heterozygous state. Jitterbug allows for identification of TEIs with an AF substantially below 0.5, such as the case of somatic mutations occurring in subclones of a tumor and therefore present in cancer cell fractions below 100 %.

Jitterbug uses as input the read alignment in BAM format of paired-end reads from the sample to the corresponding reference genome and the annotation of transposable elements in that reference genome in GFF format. We have assessed Jitterbug using simulated and real datasets and in genomes of varying complexity to benchmark different aspects of its performance. When possible we compared Jitterbug to RetroSeq, the only TEI detection software that also uses standard BAM format, and according to its authors performs better than its predecessors [24].

### Identification of homozygous TEIs in Arabidopsis using real reads and a simulated reference

In order to evaluate the performance of Jitterbug for identification of homozygous TEIs, we designed an experiment in which we should detect as insertions in a sample elements deleted from the corresponding reference. We wished to use real reads (as opposed to simulated ones) in order to more closely emulate the complexity of sequencing data and the noise in mapping signatures. For this, it is necessary to have re-sequencing data of an assembled reference genome. This is the case for the model plant *Arabidopsis thaliana,* which has a high-quality assembled genome (The Arabidopsis Genome Initiative 2000) and publicly available re-sequencing data for the reference line, Col-0 [30, 31]. In this experiment we mapped the Col-0 paired-end sequencing data to a modified reference in which 388 annotated TEs of different sizes and belonging to the different TE classes were deleted, and should thus be detected as insertions in the sample.

The raw, unfiltered results based solely on clusters of discordant reads contained a high number of false positive (FP) predictions. We evaluated the effect of mapping quality (mapQ) on the accuracy of predictions and found that poorly mapped reads (mapQ < 15) are only found in FP (Additional file 1: Figure S1), so a quality filter was implemented to exclude these reads from subsequent analyses. Even so, while sensitivity of the predictions was high at 89 % (Table 1, raw results) the positive predictive value (PPV) was still low at 37 % (Table 1, raw results). We therefore established a set of metrics aimed to discriminate true and false positives (Additional file 2: Figure S2 A) including cluster size, length of insertion interval, the span of upstream and downstream cluster and number of supporting clipped reads. As true positives and FP show different distributions (Additional file 2: Figure S2 B), we determined a set of cutoffs for each of these metrics that eliminated a large portion of the FP without excessive cost to sensitivity (Table 1, see Methods for detailed description of filtering criteria).

**Table 1:** Positive Predictive Value (PPV) and Sensitivity of Jitterbug and RetroSeq predictions in *Arabidopsis thaliana* semi*-*simulated dataset (simulated reference, real reads). In the case of RetroSeq, basepair resolution loci were extended and merged, then filtered according to the criteria recommended by the authors. For Jitterbug, the filtering was according to generated default parameters (Additional file 2: Figure S2, Methods)

		PPV (%)	Sensitivity (%)
Jitterbug	raw	37.16	89.72
	filtered	92.7	85.05
RetroSeq	extended +/- 100 bp and merged	61.01	90.26
	extended, merged and filtered	87.31	88.21

These values are a function of the characteristics of the sequencing library, and their estimation is included as a feature in Jitterbug: reasonable default parameters with which to filter the results are generated on the fly, although the user can modify them subsequently for specific purposes. By applying the described filtering criteria the resulting PPV was raised to 92 % without a substantial decrease in sensitivity (Table 1).

RetroSeq outputs single-nucleotide breakpoint positions, which tend to be found in clusters in the same vicinity, and taken as is show very poor sensitivity and specificity (data not shown). We therefore extended the predicted breakpoints to 200 bp intervals (the intervals between clusters—without relying on clipped reads—predicted by Jitterbug are on average 184 bp) and merged the resulting overlaps, then filtered them according to the author’s recommendations. The resulting sensitivity is 88 % and PPV 87 %. RetroSeq’s sensitivity is slightly better than Jitterbug’s but has lower PPV (Table 1). Since the ultimate goal is to be able to make inferences as to the impact of these polymorphisms, we prioritized optimization of PPV over sensitivity, although this depends on the goal of each study and in Jitterbug can be adjusted by the user. 93 % of the elements detected by RetroSeq were also detected by Jitterbug, which means that there is not a significant difference in the type of elements that either can detect.

Additionally, we tested whether the length of the elements was a factor in their detection by Jitterbug, and found an increase in long TEs in the false negative set (*p =* 0.0022) (Additional file 1: Figure S1 B). We also evaluated the coverage and repetitiveness at the sites of TP and FN insertions. Approximately 60 % of the false negatives can be explained by either a lack of coverage at that site, or repetitiveness of the sequence in which the insertion occurred (Additional file 1: Figure S1 C) meaning that false negatives are mostly due to characteristics of the genome itself or the sequencing run, rather than algorithmic efficiency. Since most predictions are common to both Jitterbug and RetroSeq, this holds true for both tools.

### Prediction of TEI polymorphisms in ecotypes of Arabidopsis thaliana and validation with PacBio data

One of the potential uses of Jitterbug is the identification of polymorphic TEIs within a species, for example in plant varieties different from the one for which an assembled reference is available. To assess the performance of Jitterbug in this context we have used it to identify TEIs in the *Arabidopsis thaliana* ecotype *Landsberg erecta* (Ler-1) compared to the reference *Arabidopsis thaliana* ecotype *Columbia* (Col-0). We mapped paired-end reads (180 bp fragment size, 80 bp read length) from Ler-1 [32] to the Col-0 reference sequence (TAIR10, www.arabidopsis.org). Jitterbug predicted 203 putative TEI, of these, 53 % were DNA TEs and 47 % retrotransposons.

We used publicly available Pacific Biosciences SMRT pre-assembled long reads (HGAP algorithm (Chin et al. 2013)) for the *Arabidopsis thaliana* Ler-1 ecotype (https://github.com/PacificBiosciences/DevNet/wiki/Arabidopsis-P5C3) to validate the predicted TEIs. We aligned the flanking regions (+/- 1 kb) of predicted insertions to the PacBio pre-assembled reads in order to evaluate both the PPV of the TEI predictions and the accuracy of the predicted breakpoints (see Methods for more details). Indeed, a gap in the alignment of the Col-0 sequence to the Ler-1 PacBio read confirms the presence of an inserted sequence, as well as yields information as to the length and sequence of the inserted element itself. Theoretically, the size of detectable insertions depends on the size of the Pacbio reads: for an insertion to be validated, there needs to exist a read that spans the inserted sequence and flanking regions. The length distribution of PacBio reads (Additional file 3: Figure S4) shows that 9.5 % of the reads are longer than 15,000 bp, which taken together correspond to a genome coverage of 3X. This, combined with the fact that 99.6 % of the annotated TEs in the *Arabidopsis thaliana* genome are less than 15,000 bp long indicates that there is no technical limitation to the length of detectable insertions and that each of these elements should be covered by an average of 3 PacBio reads long enough to detect the longest elements. Of the 203 predicted insertions, the anchoring regions of 190 (93 %) sites could be aligned to at least one PacBio read, while the remaining 13 sites without coverage were excluded from further analysis. Of the 190 accessible sites 180 (94.7 %) presented a median gap of more than 200 bp and for which the inserted sequence shows significant similarity (as calculated by BLAST, evalue < e-10) to a known TE sequence (Table 2) (see Methods for details on criteria for validating a TE insertion). The length distribution of the inserted sequence for validated TEI (Additional file 4: Figure S3) shows that mean size of the inserted sequences is 2800 bp, the largest inserted TE being ~14000 bp long and the smallest 216 bp.

**Table 2:** A) Summary of insertion sites independently validated with PacBio long reads for predictions generated by Jitterbug and RetroSeq. B) Comparison of validated TEI loci predicted by Jitterbug and RetroSeq

	Jitterbug	RetroSeq
Total predicted insertions (filtered)	203	622
locus aligns to PacBio	195 (96 % total)	493 (79 % total)
alignment shows insertion > 200bp	190 (93 % total)	160 (25 % total)
inserted sequence is TE	180 (88 % total)	132 (21 % total)
	confirmed insertions	
	Jitterbug (180)	RetroSeq (132)
locus nested in TE	0	90
unique to the method	146	98
common to the two	34	

Upon analyzing the inserted sequences, we determined that Jitterbug correctly predicted the TE family of the inserted element in 96 % of the cases, and of these, 80.2 % were the most similar copy within that family, as identified by BLAST (Table 3).

**Table 3:** The inserted sequences recovered from the alignments with PacBio reads were aligned with BLAST to the sequences of annotated TEs and the best hit taken as the annotated element most similar to that inserted. Table 2 describes the percentage of TEI which call the family and/or name of that best hit TE

	% confirmed insertions	
	Jitterbug (180)	RetroSeq (132)
correct TE family and TE name	77.22	N/A
correct TE family	18.92	34.09
incorrect TE family and TE name	3.89	65.9
total	100	100

The gap position in the alignment allows us to assess the accuracy of the predicted insertion site. Transposases and integrases of DNA transposons and retrotransposons usually cleave the two DNA strands at different positions, generating a staggered cut at the target site. After insertion, the repair of the single-stranded overhang on either side of the insertion will generate a target site duplication (TSD). Therefore, depending on which strand is taken as reference, either the 5′ or the 3′ end of the target site, or even the whole target site sequence, could be considered as the insertion site from a biological perspective. In the case of a TSD, both alignment methods (BWA for Illumina reads, BLAT for PacBio reads) generate an overlapped alignment (Additional file 5: Figure S9 A). Indeed, the sample contains the target site twice and reads aligning to the forward strand will clip at the 3′ end of the TSD, whereas reads aligned to the reverse strand will clip at the 5′ end of the TSD. Jitterbug reports the position with the highest softclipped support as the breakpoint, which could be either the 5′ or 3′ side, while alignment of the PacBio reads with BLAT consistently reports the 3′ end of the TSD as the breakpoint (Additional file 5: Figure S9 B). Hence, in the case of a correctly called TEI, the discrepancy between the two positions is expected to be either 0 or the length of the TSD. We assessed the distance between the breakpoint predicted by Jitterbug and that predicted by the alignment (Additional file 6: Figure S5) and found that 90 of the 186 alignments (48 %) gap exactly at the predicted breakpoint, and additional 68 are within 6 bp (totaling 84 %), while only 6 alignments gap more than 100 bp from the predicted site. Overall, the breakpoints predicted by Jitterbug were highly accurate with a median of 1 bp difference with the breakpoint predicted by the alignments. These observations are consistent with the expected distance being either 0 or the length of the TSD. In the case that there are several PacBio reads overlapping the insertion site one would expect all of the alignments to concord in the insertion site and length of the inserted element (assuming Ler-1 is truly homozygous as expected). We evaluated the consistency of breakpoints over the set of PacBio reads corresponding to each TEI locus by measuring the distance between breakpoints over the set of alignments and their standard deviation (Additional file 7: Figure S6 A). Of the 186 TEI 171 could be aligned to 3 or more reads and most of the alignments are highly consistent in their breakpoint position, with variation close to null. Predicted TEI sites greatly differing from the PacBio breakpoint tend to show a high variation between PacBio reads as well, indicating that these sites allow for multiple correct (redundant) alignment possibilities due for example to tandem or simple sequence repeats at the insertion site.

For comparison, we used RetroSeq to predict TEIs in the same dataset and validated these predictions using Ler-1 PacBio reads as described above. RetroSeq predicted 826 TEI, which we padded by 100 bp up-and downstream and merged, resulting in 622 non-redundant predicted TEI. Of these, 493 (79 %) sites align to at least one PacBio read. However, PacBio reads can be aligned without gap across the majority of the predicted TEI sites, and only 132 (21 %) of the predicted TEIs spanned by PacBio reads were validated using the same criteria used for Jitterbug (Table 2). Of these, 90 reside in an annotated TE sequence, and therefore their interpretation is ambiguous. TEs can indeed transpose as nested insertions, but from a technical point of view it is not clear to discern whether this gapped alignment is due to an inserted sequence, a mis-assembly of the reference or an ambiguous alignment to the PacBio reads (the last two cases being common in repetitive sequences), nonetheless, we counted these as correct. In order to evaluate breakpoint accuracy, we used the non-padded TEI predictions, which align to 3 or more PacBio reads (total 116) (Additional file 6 Figure S5). Only 2 correspond exactly to the breakpoint predicted by the alignments and 20 % were found to be within 6 bp, the median distance to the alignment gap position being 25 bp. The predicted TEI sites greatly differing from the PacBio breakpoint tend to show a high variation between the alignments of the PacBio reads as well (Additional file 7: Figure S6B). The larger proportion of predictions showing high variation between alignments is consistent with the fact that many reside in repeats. There was little overlap between the TEI predicted by the two tools (34 common TEI were predicted by both, Table 2), and most of the TEI unique to RetroSeq were nested in annotated TEs. We used RepeatMasker (http://www.repeatmasker.org) to assess the repetitiveness of the sequences surrounding the TEI loci predicted by the two tools and found that within a 2000 bp window around the TEI locus, 80 % of bases were masked for TEI predicted by RetroSeq, compared to 10 % for Jitterbug (Additional file 8: Figure S7).

This experiment conclusively demonstrates, without resorting to a simulation, that Jitterbug is able to identify TEI with very high PPV and that the breakpoint positions are highly accurate. Jitterbug is substantially more precise than RetroSeq in all regions (insertion sites) that can be ascertained by PacBio long read technology, i.e. that PacBio reads can be reliably aligned to.

We have made the design decision that Jitterbug only predicts TEIs in non-repetitive regions, and this restriction is partially responsible for the discrepancy between the predictions of Jitterbug and RetroSeq. This is a choice made to ensure specificity, and does not present a limitation when searching for TEI affecting phenotype as the impact of a nested TE insertion is likely minor compared to that of the already present TE. Moreover, given the currently available sequencing methods, the sensitivity and specificity in highly repetitive and centromeric regions can not be ascertained, even when using the technology providing the longest reads to date.

### Identification of heterozygous TEIs in human genomes using simulated reads

One of the key features of Jitterbug is the capacity to determine the zygosity of TEIs. This is useful in determining the prevalence of a TEI in population studies and in identifying somatic mutations in plants and animals. It is also useful in analyzing inheritance patterns in parent–child trios (a study design often used to identify causal variants in rare diseases), and estimating the clonality (cancer cell fraction) of mosaic TE insertions in cancer samples.

In order to test the ability of Jitterbug to detect heterozygous TEIs and the accuracy of the allelic frequency prediction in the human genome, we designed a simulated dataset by generating simulated reads from a modified hg19 reference sequence. This choice was made since a simulation using real re-sequencing data similar to the one described above for *Arabidopsis thaliana* was not feasible. Indeed, the simulation of heterozygous insertions requires, in addition to the assembled reference and the re-sequencing reads, an independent re-sequencing dataset from the same genotype to ensure that false positive predictions are not actually a true but un-assembled allele of the reference genome. As this combination of datasets is currently not available for human we generated a modified reference sequence from the human reference genome hg19 (limited to chromosomes 1 and 2 to reduce computation time without reducing complexity) by inserting a representative set of TE sequences at random locations, excluding already annotated TEs and regions of Ns.

Approximately half of the insertions were simulated as homozygous, and the other as heterozygous. We then simulated reads from the modified reference using DNemulator [34], which takes care to mimic the expected distribution of sequencing errors (see Methods for more details). Reads were generated at 10X, 20X and 40X coverage depths, and mapped to the original reference sequence using bwa. Though sequencing errors were taken into account, simulated reads are different from reads produced by real sequencing runs as local coverage variations, bias due to GC content and other genome-specific biases are typically not perfectly simulated. Therefore the results of this benchmark are potentially better than what is expected for real data.

**Fig. 2:**
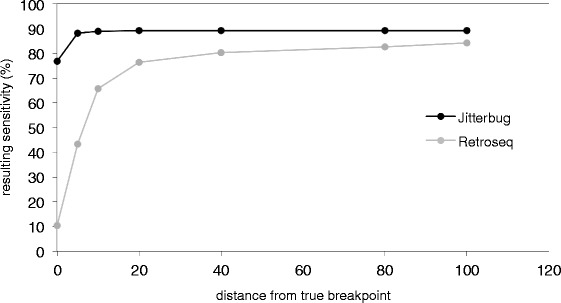
Accuracy of breakpoints predicted by Jitterbug (A) and RetroSeq (B) at 10X, 20X and 40X coverages in hg19 ND simulation. Jitterbug reaches maximum sensitivity by padding breakpoint positions by 20bp, RetroSeq reaches maximum sensitivity with 200bp padding

As in the other analyses, we ran RetroSeq as well as Jitterbug in order to compare their performance, and the predicted insertions were compared to the simulated ones in order to evaluate PPV and sensitivity (Table 4). Jitterbug shows a PPV of >99 % at all coverage levels, and sensitivity increasing with coverage from 82 % to 89 %. RetroSeq’s sensitivity also increases with coverage, from 39 % to 84 %, however the PPV decreases with increased coverage, from 98 % to 91 %. The sensitivity estimates for both tools on simulated human data are comparable to the estimates for *Arabidopsis thaliana* Col-0 using real reads, while the PPV is markedly better, an effect we attribute to the inability of simulations to reflect difficult rearrangement constellations and sequencing data biases. Jitterbug’s breakpoints are accurate within 20 bp, while RetroSeq breakpoints are accurate within 400 bp (Fig. 2, data shown for 40X). This is consistent with the previously determined breakpoint accuracy based on alignments of predicted TEI sites to PacBio reads (Additional file 7). On the 40X coverage dataset and an 8CPU, 16G RAM system, Jitterbug runs in 7 min, and RetroSeq in 3 h 40 min (Additional file 9: Figure S8).

**Table 4:** Accuracy of Jitterbug and RetroSeq at detecting homozygous (HOM) and heterozygous (HET) TEI, and predicting their zygosity, in simulated human dataset (simulated reads)

	Coverage	TP	FP	FN	PPV (%)	Sensitivity (%)	Accurate Zygosity (%)	HET detected (%)	HOM detected (%)
Jitterbug	10X	2693	1	579	99.96	82.3	94.83	78.52	86.08
	20X	2825	0	447	100	86.34	99.58	84.58	88.10
	40X	2919	0	353	100	89.21	100	88.25	90.17
RetroSeq	10X	1308	23	1964	98.27	39.98	-	8.51	71.37
	20X	2528	115	744	95.56	77.26	-	72.28	82.23
	40X	2754	258	518	91.43	84.17	-	81.70	86.63

**Fig. 3:**
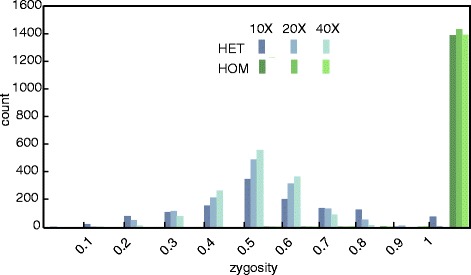
Distribution of predicted zygosity for heterozygous (HET) and homozygous (HOM) insertions, at 10X, 20X and 40X coverage in hg19 ND simulation. Homozygous insertions are consistently (>94 % ) predicted as such (zygosity = 1). The zygosity of heterozygous insertions is a distribution centered around 0.5, the correct frequency, which narrows with increasing coverage

This simulation includes both heterozygous and homozygous insertions, and Jitterbug predicts the correct zygosity in nearly all cases (95 % at lowest and 100 % at highest coverage) (Table 4) with the predicted allelic frequency of heterozygous insertions following a distribution centered around 0.5, which narrows with increasing coverage (Fig. 3). RetroSeq is expected to estimate the zygosity of insertions in a later version, however personal communication with the author confirmed that this option is not yet implemented. Therefore we were not able to compare the performance of zygozity estimation in this context. We conclude that, for Jitterbug, genome size and complexity has no measurable effect on TEI prediction accuracy in unique regions of the genome, that heterozygous TEIs can be readily detected and zygosity of TEIs can be accurately predicted given adequate coverage.

### Prediction and experimental validation of TEIs in a 1000 Genomes Project trio

Testing the performance of NGS-based variant predictions in real human data is hampered by the availability of a gold standard dataset. However, experimental validation of predictions is available for some individuals from the 1000 Genome Project (1000GP, http://1000genomes.org) that have been studied by deep sequencing. Here we compare the results of Jitterbug with PCR-validated TEIs that were previously predicted in various 1000GP samples.

Hormozdiari and collaborators [25, 26] predicted TEIs in 8 individuals from the 1000GP dataset using VariationHunter, specifically looking for Alu insertions. These consisted of one trio from Yoruba (YRI, NA18506, NA18507, NA18508), one individual from the CEU population (NA10851), one from Korea (AK1), one Han Chinese (YH) and two from Khoisan (KB1 and HGDP01029). Amongst these individuals 35 sites (a site corresponds to an insertion at a given locus in one or more individuals) were chosen for experimental verification by PCR amplification, totaling 95 experimentally determined insertions. This allowed us to determine both FP (unvalidated predictions) and FN (absence of a validated insertion in an individual at a site predicted in another) for both Jitterbug and VariationHunter. Jitterbug outperforms VariationHunter in both PPV and sensitivity over the 95 experimentally validated insertions (Table 5). Furthermore, 29 sites, corresponding to 69 insertions, were selected in the YRI trio to assess zygosity by PCR using allele-specific primer pairs to detect both the insertion and the reference allele. Jitterbug correctly predicts the zygosity in all cases, showing that the high accuracy Jitterbug achieved on simulated human data is preserved with real data.

**Table 5:** Comparison of Positive Predictive Value (PPV) and Sensitivity of PCR-validated TEI for Jitterbug and VariationHunter in 1000 GP samples analyzed in Hormozdiari et al. 2011

	PPV (%)	Sensitivity (%)
VariationHunter	53.33	88.89
Jitterbug	64.29	90.00

### Identification of TEI in subclonal fractions of tumors using simulated reads

Identifying somatic TEIs as those found in cancer samples holds specific challenges, which we have addressed with Jitterbug. The first challenge being that tumor samples are often collected from heterogeneous tissues, and therefore TEIs might be found in low frequency in the sample. As these events have weak alignment signatures (a small fraction of reads from that locus predicts the insertion), they can be more difficult to identify and to distinguish from background noise. The second challenge is properly exploiting pairs of matched tumor and normal sequenced samples (from here on TD and ND, respectively) to correctly distinguish true somatic insertions in the tumor sample from germline TEIs that have been missed by the TEI prediction in ND, by assessing the likelihood that the TEI exists in the normal tissue.

**Fig. 4:**
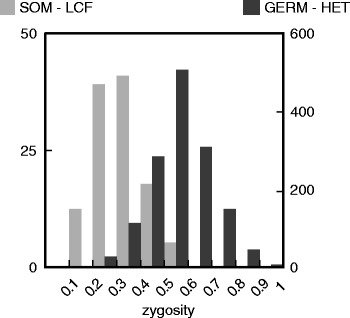
Distribution of predicted zygosity for germline heterezygous (GERM-HET) and somatic low cell frequency (SOM-LCF) insertions in simulated TD sample at 40X coverage. These distributions are centered around the correct frequencies: 0.5 and 0.25, respectively

We generated a simulated tumor-normal dataset using the simulation in the human genome described above (with a total of 1634 homozygous insertions, and 1638 heterozygous insertions, see Table 4) as ND. The TD sample was then simulated by adding 73 insertions at 25 % allelic frequency to the same modified reference to simulate the case of low cell fraction TEI (LCF-TEI). Reads were generated at 10X, 20X and 40X for the simulated TD sample as described above. We identified TEI in the simulated TD sample with Jitterbug, as well as with RetroSeq for comparison. Both Jitterbug and RetroSeq were able to recover around 90 % of LCF-TEIs at 20X and higher coverage, however only Jitterbug was able to recover a fraction (42 %) of these at low (10X) coverage (Table 6). Jitterbug predicts the allelic frequency of these insertions as a distribution centered around 0.25, the expected frequency (Fig. 4, distribution shown for 40X coverage).

**Table 6:** Percentage of low-frequency TEI (LF_TEI) detected by Jitterbug and RetroSeq at various coverages

	Coverage	LCF TEI detected (%)
Jitterbug	10X	42.47
	20X	89.04
	40X	89.04
RetroSeq	10X	0.00
	20X	89.04
	40X	90.41

### Distinguishing somatic and germline TEIs using simulated matched tumor-normal pairs

The majority of TEIs called in a patient TD sample are expected to already be present in the matched normal (ND) genome, therefore true tumor somatic TEIs can only be called by comparison to the ND sample results. Furthermore, there is an overlap between the allelic frequency distributions of the somatic LCF-TEI and the heterozygous germline TEI (GERM-HET) **(**Fig. 4), thus low frequency TEI cannot be called as somatic simply based on their low allelic frequency.

The commonly applied strategy for identification of tumor-specific structural variations is based on comparing the set of predictions in a TD and its matched ND sample, retaining as putative tumor-specific variations those that are unique to the TD sample. However, these might be falsely called as tumor-specific if the corresponding insertion in the ND sample was a false negative (FN) due to, for example, local low coverage. To correctly call somatic TEI and discard germline TEI, one must exploit the alignment information supplied by the matched normal analysis, even in regions where a TEI has been called in TD but not in ND, in order to avoid that a false negative in ND leads to a false positive somatic TEI prediction. As discordant reads are indicative of a TE insertion, the presence of such reads in the normal sample at the locus where a TEI was predicted in the TD sample might enable us to discern FN from true negatives (TN), thus enabling to classify insertions unique to the TD sample as germline or somatic, respectively. Similarly, low coverage in the ND sample could indicate a FN at that locus. We have implemented a module that performs this comparison and examines the genomic location of the putative tumor-specific insertions for coverage and presence of discordant reads in the ND sample.

**Fig. 5:**
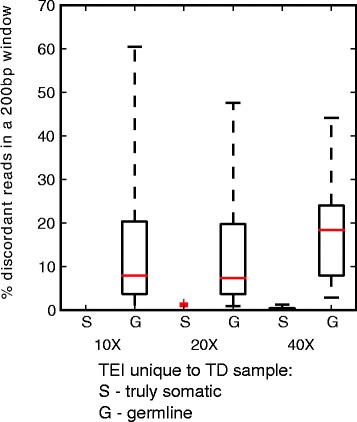
Boxplots representing the percentage of discordant reads found in a 200bp window around the insertion site of putative somatic insertions (unique to TD sample). At 40X coverage, it is possible to discriminate between the truly somatic ones (S) and the germline ones (G), at lower coverage it is possible to distinguish S and G to a large extent

We have tested this module on the simulated ND/TD pair described above. Of the insertions unique to the TD sample, some are truly somatic and others are germline insertions, but were not identified in ND, corresponding to FN. Consistent with previous results the number of FN decreases with coverage, as does the number of incorrectly called tumor-specific somatic insertions. We plotted the percentage of discordant reads found in a 400 bp window around the insertion site in the ND sample, for both the true somatic insertions (S) and the germline TEI falsely called as somatic (G) **(**Fig. 5). The fraction of discordant reads is consistently higher in the germline insertions falsely called as somatic than in the true somatic ones. Using a cutoff of minimum 2 % discordant reads to call an FN in ND, one can discard 100 % of the germline predictions at 40X without losing any true somatic ones (Table 7). This criterion is useful at all coverage levels, e.g. discarding 90 % of germline predictions at 10X. Most cancer genomes are sequenced at greater than 30X coverage, meaning that the discriminative power of combined tumor/normal comparison followed by FN identification and filtering according to discordant read percentage at the corresponding ND locus is highly reliable. RetroSeq does not provide the functionality of distinguishing somatic and germline TEIs based on TD/ND pairs and could thus not be compared for this purpose.

**Table 7:** Percentage of truly somatic (S) and germline (G) insertions, among the insertions predicted in the TD but not the ND sample, eliminated according to various cutoff values. The cutoff corresponds to the percentage of discordant reads in a 400bp window around the TEI and is used to determine the presence of a FN in the ND sample

			% predictions eliminated			
	10X		20X		40X	
Discordant read cutoff	S	G	S	G	S	G
> 1%	0.00	97.73	6.15	98.17	1.54	100.00
> 1.5%	0.00	95.45	4.62	95.41	0.00	100.00
> 2%	0.00	90.91	0.00	94.50	0.00	100.00
> 2.5%	0.00	83.33	0.00	86.24	0.00	100.00
> 3%	0.00	79.55	0.00	81.65	0.00	93.94

## Conclusion

Jitterbug addresses the increasingly evident need of including transposable element analysis into standard structural variation studies based on NGS. Jitterbug is an accurate, fast and user-friendly tool to predict TEI using mapping signatures of paired-end sequences and clipped reads, requiring only a BAM file and a GFF annotation of TEs in the reference genome. Jitterbug can be run either on a cluster or a local workstation, and is parallelizable according to the computational resources available. It has been designed to call either germline TEI in a single sample or exploit tumor/normal paired datasets to identify somatic insertions. It is able to detect low-frequency insertions as those found in heterogeneous tissue or tumor samples and predicts the zygosity and/or variant allele frequency of the insertion. Finally, the fact that Jitterbug uses genome annotations to define potentially mobile sequences makes it adaptable to other analyses such as searching for insertions of viruses, reporter constructs or other types of sequences.

We have extensively tested Jitterbug on both simulated and real datasets with independent validations based on PacBio sequencing as well as PCR, and conclude that we can predict TEI with high sensitivity and PPV, accurately determine their allelic frequency and are able to correctly call somatic insertions in paired tumor/normal datasets at low cancer cell fraction. We benchmarked it against RetroSeq, chosen as it also runs in the standard BAM format. Jitterbug does outperform RetroSeq in all the experiments, and offers additional features such as exploiting clipped-reads, zygosity prediction and processing matched tumor/normal pairs. However it is worth noting that their performance was most similar on fully simulated datasets (simulated reads) and diverged most when using real data validated by PacBio. This highlights the fact that simulated datasets cannot fully represent the constellation of noise and variants present in a true biological sample, and that independent validation is necessary to correctly assess the sensitivity and PPV of an algorithm. We suggest the need to develop an independently validated, gold-standard dataset for benchmarking as a necessary resource for the development of SV detection algorithms, and consider the PacBio-based evaluation for *Arabidopsis thaliana* Ler-1 TEIs developed in this study as a first step in this direction. Looking forward, we are actively developing Jitterbug to utilize split-read signatures such as those generated by bwa-mem, thus improving performance at low coverage or for inserted elements that are smaller than the read length (as would be the case for MITEs, for example).

## Methods

### Overview of the Jitterbug approach

The algorithm follows the following main steps:

0. Calculate mean and standard deviation of insert size (fragment length) and read length over 1,000,000 properly paired read pairs (alternatively, a config file can be provided with these metrics).
1. Select “valid” discordant reads from the BAM file. For this, scan the bam file and reject any read pair that is flagged as “proper pair” (SAM bitwise tag 0x2), or that has a mapping distance less than the expected insert size, or where both reads in a pair are mapped repetitively.
2. Of the valid discordant pairs, select those that have one read mapping uniquely to a non-TE location (“anchor” read), and the other read mapping (repetitively or not) to at least one location that is annotated as a TE in the provided annotation (“TE mate” read).
3. Cluster anchor reads according to the overlap of their predicted insertion interval, on the forward and the reverse strand.
4. Forward and reverse clusters are paired if their predicted insertion intervals overlap. Each cluster pair calls one putative TE insertion, with the insertion site falling within the intersection of the forward and reverse predicted intervals. Clipped reads are retrieved for that interval in order to calculate the exact breakpoint, and properly mapped reads, which span this breakpoint are tallied. The ratio of clipped to (clipped + spanning) reads is used to estimate the variant allele frequency (VAF).
5. Final results are written as a GFF file annotating the insertion sites, and a table file describing the clusters and reads that compose each prediction, meant to be easily manipulated with standard *NIX tools in order to extract more detailed information such as the read sequences, useful for designing PCR primers.
6. GFF files can be filtered using the default filtering criteria supplied in a configuration file, or according to the user’s preferences.

### Detection of TEIs using paired-end and clipped short reads

The Jitterbug tool is implemented in Python (www.python.org) using the pysam library (https://github.com/pysam-developers/pysam) to process BAM files. Discordant read pairs were selected as read pairs where

$$ mapping\  distance > 2* fragement\  length $$

or where the two reads mapped to different chromosomes. Valid discordant pairs are those that have one read mapping non-ambiguously to a non-TE location (the “anchor”), and the other mapping to a TE in one or more of its annotated mapping positions (the “TE mate”) (alternate positions recorded in the XA tag of the BAM file, see http://samtools.github.io/hts-specs/SAMv1.pdf). The mapping quality filter is applied to the anchor read only, as most aligners always attribute the lowest score to repetitively mapping reads. Each valid discordant read pair predicts a TE insertion in an interval calculated as:

$$ anchor\  start\  position + fragment\  length + s* sdev\left( fragment\  length\right) $$

for anchor reads mapped to the forward strand, and

$$ anchor\  end\  position\ \hbox{--}\ \left( fragment\  length + s* sdev\left( fragment\  length\right)\right) $$

for anchor reads mapped to the reverse strand.

Anchor reads are clustered by finding the set of maximal overlapping intervals, i.e. sets of reads for which all intervals are overlapping. Overlapping clusters themselves are then discarded, to retain only isolated clusters. Forward and reverse clusters are paired if their respective intervals overlap. These steps are parallelized by bins, the size of which can be set by the user, or by chromosome, if multiple processors are specified and no bin size is set, using the Python multiprocessing library (https://docs.python.org/2/library/multiprocessing.html). It is advised not to set the bin size too small, as a TEI can be missed if its forward and reverse cluster end up in separate bins.

The insertion site interval is bounded by the greatest start position of the set of reads in the forward cluster, and the smallest end position of all reads in the reverse cluster. This interval is further narrowed down if any reads found within this interval are clipped. Support for this clipped position is calculated as the number of reads that are clipped at the same (+/- 3 bp) position.

### Zygozity estimation using reference-like and clipped reads

If the exact position for a TEI has been determined by clipped read signature, the original bam file is queried for all reads that overlap this position. Those that are properly mapped (bitwise flag 0x2 in SAM specification) and overlap the insertion site with five or more nucleotides on each side (termed core-reads) indicate the presence of a reference allele. For each given TEI, zygosity (or variant allele frequency) is calculated as

$$ \frac{clippedreads}{clippedreads+ corereads} $$

### TEI filter optimization

We established a set of metrics according to which TEI predictions can be evaluated. These metrics are:

- Cluster size: number of reads in the forward and reverse strand clusters
- Span: maximum distance between the start positions of reads in a cluster. A span of 0 means the reads are stacked.
- Clipped support: Number of clipped reads supporting the same insertion position within the predicted insertion interval
- Interval length: length of the predicted insertion interval, calculated as the distance between the start position of the innermost read in the forward cluster, and the end position of the innermost read in the reverse cluster.
- Consistent TE: whether TE mate reads of both forward and the reverse clusters map to the same annotated element.

For the simulation in the *Arabidopsis thaliana* genome using real Col-0 reads, we plotted the TP and FP according to these metrics, and were able to determine cutoffs for each of these criteria, which optimize PPV and sensitivity. These cutoffs are:

$$ 2 < cluster\_ size < \left( 5* coverage\right) $$

$$ 2 < span < mean\_ fragment\_ length $$

$$ \begin{array}{l} mean\_ read\_ length < interval\_ length < 2\ *\ \Big( mean\_ fragment\_ length + 2* sdev\_ fragment\_ length\ \hbox{--} \\ {}\left( mean\_ read\_ length\ \hbox{--}\ sdev\_ read\_ length\right)\Big)\end{array} $$

$$ 2 < clipped\_ support < \left(5* coverage\right) $$

### Identification of somatic TEIs

The identification of somatic TEIs using tumor and normal samples (TD and ND) is conducted in two steps. First, TEIs are predicted with the main Jitterbug module in each sample separately, and the TD results are quality filtered. The insertions present in the filtered TD set and absent from the unfiltered ND set are retained as putative somatic insertions. For each of these, the reads within a 200 bp window around the corresponding locus in the ND BAM file are extracted and the counts of discordant and concordant mapped reads are tallied. Comparing true to false somatic TEIs in the simulation we found that 2 % or more discordant reads at that locus in ND indicates an FN in ND and the insertion is not called as somatic. Furthermore if the average coverage within the 200 bp window is below 8X we assume a FN in ND. Thus, only the insertions that are unique to the TD sample, have sufficient coverage and close to no discordant reads at the corresponding locus in the ND sample are retained as somatic. These steps are all implemented in a separate module, which takes as input the unfiltered predictions from TD and ND, the ND BAM file and the filtering parameter configuration file generated by Jitterbug when run on the TD sample.

### Primer design to verify insertions and sequence inserted element

To verify the presence of the predicted inserted element, one can design primers against the sequence flanking the insertion site, and one primer within the inserted TE. In order to amplify the entire inserted element, one can design primers upstream and downstream of the insertion site – these will yield a short amplicon (their distance in the reference) in the absence of an insertion, and a long amplicon (or none, depending on the conditions) in the case of an insertion. To do this, it is best to locally assemble the reads covering these regions, as SNPs or short variants with respect to the reference that would not prevent read mapping might impede primer binding. One can extract from the .table output file the sequence for the anchor and TE mate, for the forward and reverse cluster using standard *NIX tools grep and cut (see web documentation for more details and example script).

### Simulation of TEIs in the Arabidopsis thaliana Col-0 reference genome

The *Arabidopsis thaliana* reference genome (TAIR10) was downloaded from www.arabidopsis.org. 388 TEs were randomly selected from the sets of TEs annotated as retroelements and DNA transposons, thus covering equally the two classes of TEs found in this genome. The elements were selected randomly over the set of annotated elements in order to get a distribution in size of the elements (excluding annotated fragments smaller than 200 bp). These were cut-and-paste into random locations in the genome (excluding regions within 100 bp of already annotated TEs and Ns). The script used to perform this simulation and lift over annotations to the modified sequence is available at https://sourceforge.net/projects/kitchen-drawer/files/sim_SV.py/download. Elements that were deleted from this reference should then be detected as insertions in the resequencing data of the reference strain Col-0, and the positions of the deleted elements have been used to benchmark the predictions of Jitterbug and RetroSeq.

### Simulation of TEIs and Illumina reads for the human reference hg19

The human reference genome (hg19) was downloaded from www.ucsc.edu and only chromosomes 1 and 2 were used for the following simulations. The script used to simulate TE movement is the same as mentioned above.

In order to simulate the ND sample with both heterozygous and homozygous TE insertions, we generated a simulated reference as two “alleles”. First, 1634 TEs were selected randomly from the annotated TEs (excluding fragments smaller than 200 bp) and cut-and-paste into random locations (excluding regions within 100 bp of already annotated TEs and Ns), to generate a modified reference (hg19_mref1) containing what will be the homozygous insertions. We then selected another 1638 TEs to be cut-and-paste into random locations (according to the same criteria as previous step) in a duplicate of hg19_mref1, generating hg19_mref2. Taken together, hg19_mref1 and hg19_mref2 represent the two “alleles” of the modified reference, containing 1634 simulated TEI present in two copies (homozygous), and 1638 TEI present in only one (heterozygous). We then generated reads from both hg19_mref1 and hg19_mref2 at 5X, 10X and 20X depth of coverage, which combined yield a total coverage of 10X, 20X and 40X, respectively. The dataset for each depth of coverage thus contains reads corresponding to the homozygous and heterozygous TEI.

In order to simulate the TD sample, we added to the ND genome 73 TEI at a final allelic frequency of 25 %. For this, we took the “allele” hg19_mref2 and generated a third allele, hg19_mref3, by adding 73 TE to this sequence, in the same way as described above. We then took as reference four alleles: two copies of hg19_mref1, one copy of hg19_mref2 and one copy of hg19_mref3. Thus the homozygous TEI described previously are still homozygous (present in all four), the heterozygous TEI are still heterozygous (present in mref2 and mref3) and the low-frequency tumor TEI are present at 25 % allelic frequency (present in mref3 only). We then generated reads from both copies of hg19_mref1, hg19_mref2 and hg19_mref3 at 2.5X, 5X and 10X coverage, which combined yield a total coverage of 10X, 20X and 40X, respectively. The dataset for each depth-of-coverage thus contains reads which correspond to the homozygous, heterozygous and low-cell-fraction tumor TEI.

Reads were simulated from the modified reference sequences using the DNemulator package (www.cbrc.jp/dnemulator/) [34] and fastq files from the 1000 Genomes Project sample NA18506 (http://www.ncbi.nlm.nih.gov/sra/ERX009608) as a model for sequencing errors, with fragment length 450 +/- 40 bp and read length 100 bp. As the DNemulator package generates read names that include the position from which the read originates, reads in a pair do not have identical names, which RetroSeq and Jitterbug both rely on. Therefore the names of the simulated reads were modified so that both reads in a pair had identical names. The reads were mapped to chromosomes 1 and 2 of hg19 using bwa (aln -n 4 -o 1 -e 3).

### Analysis of simulated tumor-normal pairs

The simulated TD and ND samples described above were analyzed as pairs at 10X, 20X and 40X depth of coverage. We used the TE annotation for the hg19 reference sequence provided by UCSC table browser (http://genome.ucsc.edu/cgi-bin/hgTables) and curated to add a tag of the form “Name = FAMILY_x” where FAMILY is the name of the element’s family, and x is a digit, thus generating a unique name tag which indicates the TE family name. The final annotation used is available on http://public-docs.crg.es/sossowski/jitterbug/. The mapped reads in BAM format for either sample and TE annotation were supplied to the compare_ND_TD module, which runs the main Jitterbug module on both sample BAMs (bin size set to 50,000,000 bp), filters the results according the default values generated on the fly, and calls somatic TEs as explained above.

### Detection of TEIs in the Arabidopsis thaliana Ler-1 strain

Paired-end sequencing data for the Ler-1 strain was obtained from [32] and mapped to the TAIR10 reference genome (www.arabidopsis.org) using bwa (aln -n 4 -o 1 -e 3). The library characteristics are: fragment length 457.98 +/- 51.08 bp, read length 78.25 +/- 2.71 bp (quality trimmed), coverage 38X.

The annotated TEs were extracted from the TAIR10 annotation (ftp://ftp.arabidopsis.org/home/tair/Genes/TAIR10_genome_release/TAIR10_gff3) using “grep transpo” to select annotations of type:

$$ \left[\mathrm{transposon}\_\mathrm{fragment}\left|\mathrm{transposable}\_\mathrm{element}\right|\mathrm{transposable}\_\mathrm{element}\_\mathrm{gene}\right] $$

This annotation was further curated to merge overlapping elements, include MITEs annotated by [4] and to add a tag to the 9th column of the form “Name = FAMILY_x” where FAMILY is the name of the element’s family, and x is a digit, thus generating a unique name tag which indicates the TE family name. The final annotation is available on http://sourceforge.net/projects/jitterbug/data. Jitterbug was run (bin size set to 1000000) and results were filtered using generated default parameters.

### Validation of Ler-1 TEIs using long reads from PacBio-SMRT

The set of 212,997 PacBio HGAP-preassembled reads (mean length 9814 +/- 4138 bp) were downloaded from the Pacific Biosciences public data repository (https://github.com/PacificBiosciences/DevNet/wiki/Arabidopsis-P5C3). The sequences flanking the insertions (2000 bp window) predicted by Jitterbug and RetroSeq were extracted from the *Arabidopsis thaliana* Col-0 reference sequence (TAIR10, www.arabidopsis.org) using the Bedtools tool fastaFromBed [35]. These sequences were aligned to the set of PacBio reads using BLAT [36] with default parameters. Alignments were filtered using pslcDNAfilter from the BLAT suite to extract alignments with 97 % minimum identity and 30 % minimum query coverage. An in-house tool was developed to chain alignments ordered along the same query and same target, collapsing them to contiguous aligned segments. The first criterion to validate a predicted TEI is that the median gap size across the grouped alignments is longer than 200 bp. As the PacBio data is high-coverage (17X) one would expect several reads to overlap the insertion site, and all of the alignments to concord in the insertion site and length of the inserted element (assuming Ler-1 is truly homozygous as expected), so the second criteria is that the mean gap size standard deviation is less than half the length of the TE size.

### Detection and validation of TEIs in a human 1000GP trio

The raw read data were downloaded for a mother, father and male child trio from Yoruba, Nigeria (YRI).

Mother: NA18508 - Exp ERX009610 (http://www.ncbi.nlm.nih.gov/sra/ERX009610)

Father: NA18507 - Exp ERX009609 (http://www.ncbi.nlm.nih.gov/sra/ERX009609)

Child: NA18506 - Exp ERX009608 (http://www.ncbi.nlm.nih.gov/sra/ERX009608)

These sequencing libraries are of 300 bp fragments, 100 bp paired reads. Reads were aligned with bwa (aln -n 5 -o 1 -e 5) to the hg19 human reference genome. The TE annotation used was that described previously in the Methods section “Analysis of simulated tumor-normal pairs”. Jitterbug was run (-b 50000000 -q 15) and results were filtered according to generated parameters. The predicted insertions were compared to the PCR-verified insertion sites found in the supplementary material of Hormozdiari et al 2011, downloaded from the supplementary material tables available (http://genome.cshlp.org/content/suppl/2010/12/03/gr.115956.110.DC1/Hormozdiari115956_Supplementary_Tables.xls). For each of the sites verified by PCR, we checked whether it was also predicted by Jitterbug, and thus tallied TP, TN, FP and FN. The sites verified by Hormozdiari et al 2011 came from those identified by VariationHunter in any of the 8 individuals analyzed in this paper, and in some cases, these had not been predicted computationally but were identified experimentally. Thus, we were also able to calculate PPV and sensitivity for VariationHunter over these sites.

### Running RetroSeq

RetroSeq was downloaded from https://github.com/tk2/RetroSeq and run with default parameters and results filtered according to author’s recommendations. As the predicted breakpoints are at a given nucleotide, but do not concord with actual breakpoints unless padded (See Fig. 2) by +/-200 bp, all predictions generated by RetroSeq were extended 200 bp up- and down-stream. Also, RetroSeq can predict several insertions at the same site but with different predicted elements, and these were merged to a non-redundant set.

### Data and software availability

The simulated data generated for these analyses, as well as the curated transposable element annotations for the human hg19 assembly and Arabidopsis TAIR10 assembly, are available on the CRG document server (http://public-docs.crg.es/sossowski/jitterbug/). All other data used were downloaded from public repositories as described.

Jitterbug is made public as open-source software under the MIT license, available at http://sourceforge.net/projects/jitterbug/. To clone the release version of the software corresponding to this publication, you can do the following: git clone git://git.code.sf.net/p/jitterbug/code jitterbug-code git checkout tags/v1.0. The mandatory input files required by Jitterbug are a BAM file of reads mapped to the reference genome and the annotation of transposable elements in that reference in GFF format. Three commands are sufficient to run Jitterbug, filter the results and compare a tumor-normal sample pair. Please see the project page on SourceForge for more usage details.

## Ethics

All human data used were obtained through the 1000GP and used according to the rules stated at http://www.1000genomes.org/about#ProjectSamples. No new human samples or data have been used that require ethical approval or informed consent.

## Additional files

Additional file 1: Figure S1. A) Influence of read mapping quality in false discovery. B) Distribution of the length of the TEs selected to generate the simulated TEI. Longer sequences are over-represented in the False Negatives (*p* = 0.002). C) Sequence context of false negatives (FN). About 60 % of false negatives can be attributed to lack of coverage or repetitive context. (ZIP 45 kb) Additional file 2: Figure S2. A) Details of Jitterbug’s prediction method with the metrics used as filtering criteria highlighted in orange. B) True Positive (TP) and False Positive (FP) predictions plotted according to these metrics. For each metric, TP and FP follow different distributions and thresholds can be determined to eliminate FP without excessive loss of TP. (ZIP 55 kb) Additional file 3: Figure S4. Distribution of the length of pre-assembled Ler-1 PacBio reads. (PDF 10 kb) Additional file 4: Figure S3. Distribution of the length of TE insertions identified in Landsberg erecta (Ler-1) compared to Columbia-0, as determined by alignment of predicted TEI loci with Ler-1 PacBio reads. (PDF 48 kb) Additional file 5: Figure S9. A) characteristic signature of Illumina short read mapping in the case of a target site duplication generated by the insertion of a mobile element. B) Example of a TEI with TSD in Ler-1 compared to Columbia-0: IGV screenshots and BLAT alignments of Illumina and PacBio sequences, mapped to the reference, respectively. Jitterbug calls the breakpoint as the position with highest softclipped reads support, which can be either side of the TSD, 5’ in the first example and 3’ in the second. BLAT reports the 3’ position on the forward (reference) strand as the breakpoint. The difference in breakpoint position determined by these two methods in many cases corresponds to either zero or the length of the TSD. (PDF 15 kb) Additional file 6: Figure S5. Fraction of total TEI compared to the distance between the predicted breakpoint and that determined by alignment of the sequences flanking the TEI loci to PacBio reads, for Jitterbug and RetroSeq (ZIP 27 kb) Additional file 7: Figure S6. For TEI which align to > 3 PacBio reads, the standard deviation of the distances between the predicted breakpoint and that determined by the alignment for each read was plotted against the mean. A deviation of 0 indicates that the same breakpoint is predicted in all alignments. A) Jitterbug: the cluster of points around 0, 0 indicate that most alignments are highly concordant between the set of reads and are close to the predicted breakpoint. B) RetroSeq: the spread of points is consistent with the fact that most TEI are predicted in annotatated TEs, which by their repetitive structure would allow multiple possible alignments. (PDF 19 kb) Additional file 8: Figure S7. Percent of TEI flanking sequences masked by RepeatMasker. On average, 80 % of the sequences flanking RetroSeq TEI (red) are masked, compared to 10 % for Jitterbug (black). (PDF 19 kb) Additional file 9: Figure S8. Runtime benchmark (hour:min:sec) of Jitterbug and RetroSeq on the simulated ND sample in hg19 (limited to chromosomes 1 and 2), at various coverage depths. (PDF 138 kb)

## Abbreviations

TEI: Transposable Element Insertion
PPV: Positive Predictive Value
PCR: Polymerase Chain Reaction
NGS: Next Generation Sequencing
TE: Transposable Element
BAC: Bacterial Artificial Chromosome
CNV: Copy Number Variation
SNP: Single Nucleotide Polymorphism
LTR: Long Terminal Repeat
AF: Allelic Frequency (within one sample, NOT within a population)
FP: False Positive
TP: True Positive
TN: True Negative
FN: False Negative
LCF-TEI: Low-Cell Fraction TEI
HET-TEI: Heterozygous TEI
TD: Tumor sample
ND: Normal sample
