# Supplementary figures and images for: Jitterbug: somatic and germline transposon insertion detection at single-nucleotide resolution
Source: BMC Genomics. 2015 Oct 12;16:768. doi: 10.1186/s12864-015-1975-5 (PMC4603299; doi:10.1186/s12864-015-1975-5)

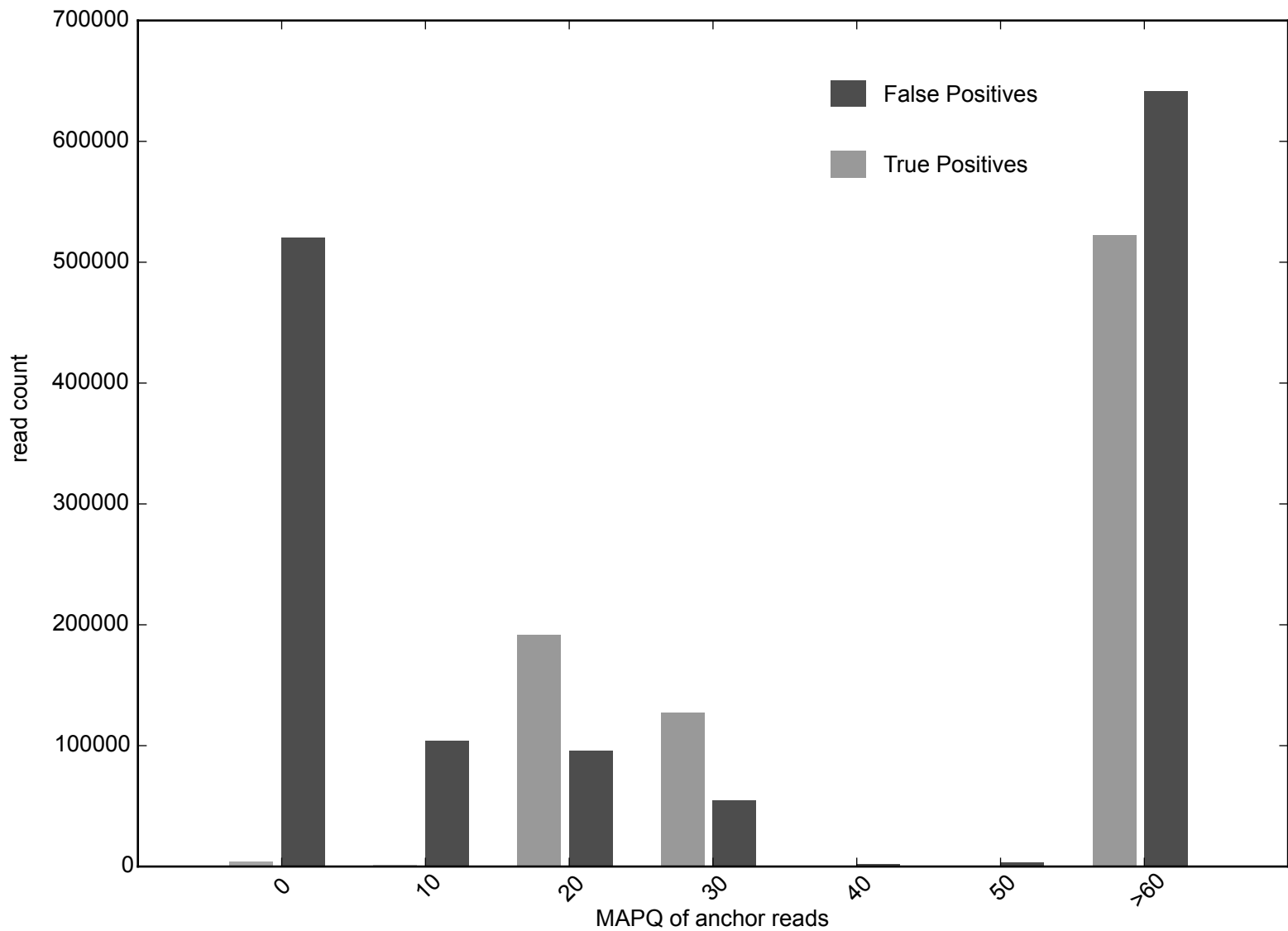

Supplement: Additional file 1: Figure S1. — A) Influence of read mapping quality in false discovery. B) Distribution of the length of the TEs selected to generate the simulated TEI. Longer sequences are over-represented in the False Negatives (p = 0.002). C) Sequence context of false negatives (FN). About 60 % of false negatives can be attributed to lack of coverage or repetitive context. (ZIP 45 kb) [file 12864_2015_1975_MOESM1_ESM.zip › add 1/Supp1A.pdf]

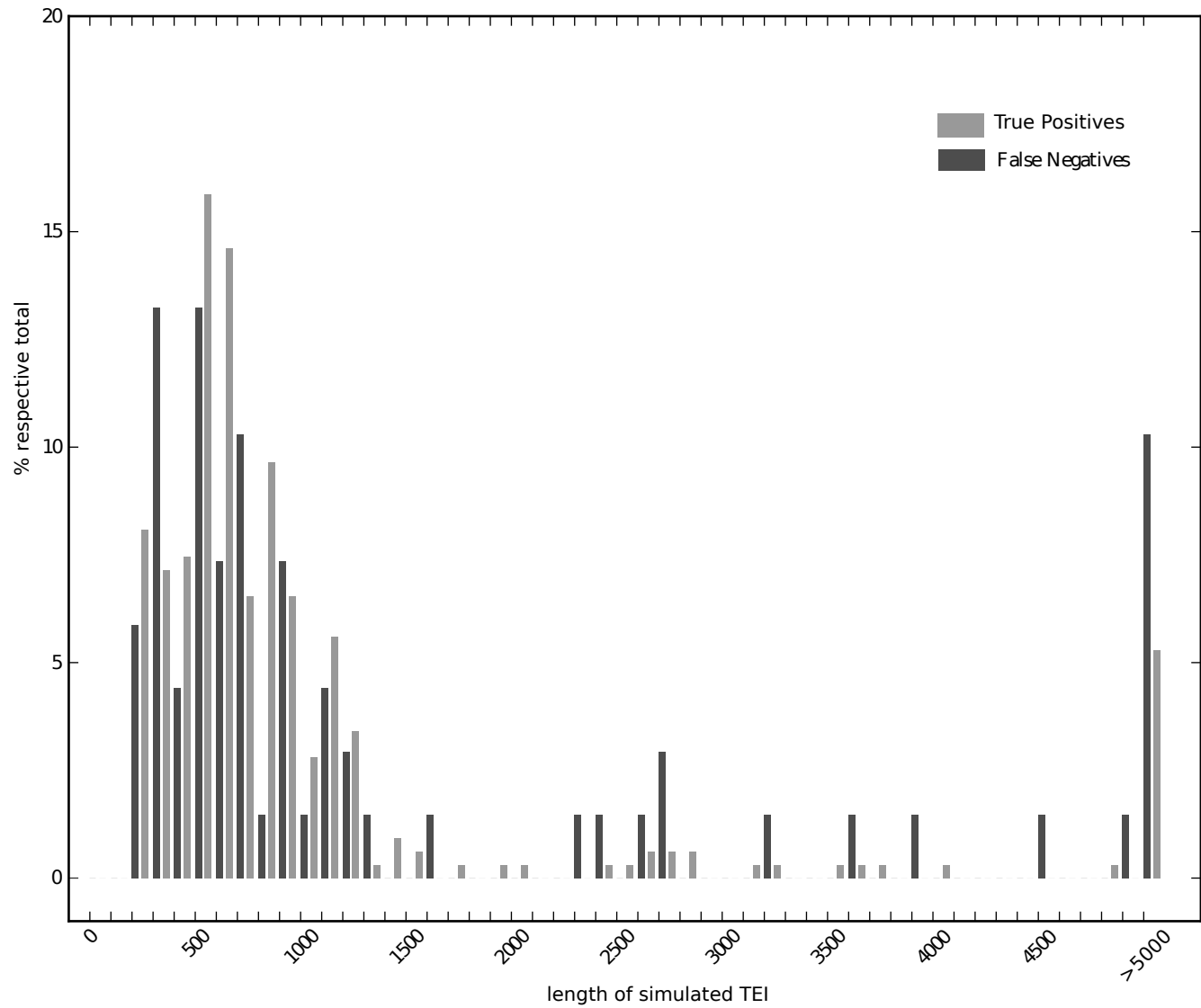

Supplement: Additional file 1: Figure S1. — A) Influence of read mapping quality in false discovery. B) Distribution of the length of the TEs selected to generate the simulated TEI. Longer sequences are over-represented in the False Negatives (p = 0.002). C) Sequence context of false negatives (FN). About 60 % of false negatives can be attributed to lack of coverage or repetitive context. (ZIP 45 kb) [file 12864_2015_1975_MOESM1_ESM.zip › add 1/Supp1B.pdf]

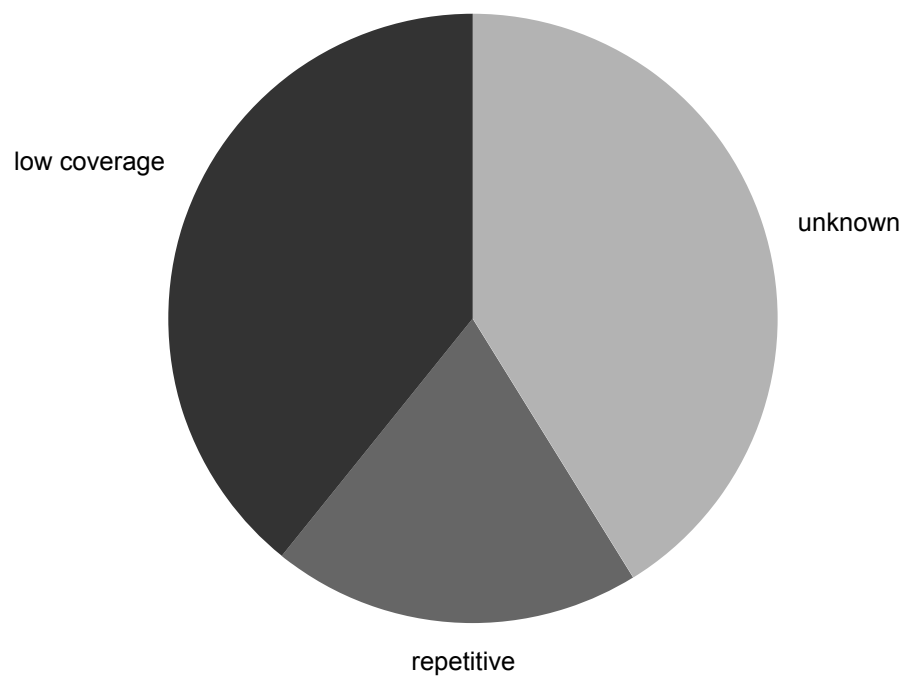

Supplement: Additional file 1: Figure S1. — A) Influence of read mapping quality in false discovery. B) Distribution of the length of the TEs selected to generate the simulated TEI. Longer sequences are over-represented in the False Negatives (p = 0.002). C) Sequence context of false negatives (FN). About 60 % of false negatives can be attributed to lack of coverage or repetitive context. (ZIP 45 kb) [file 12864_2015_1975_MOESM1_ESM.zip › add 1/Supp1C.pdf]

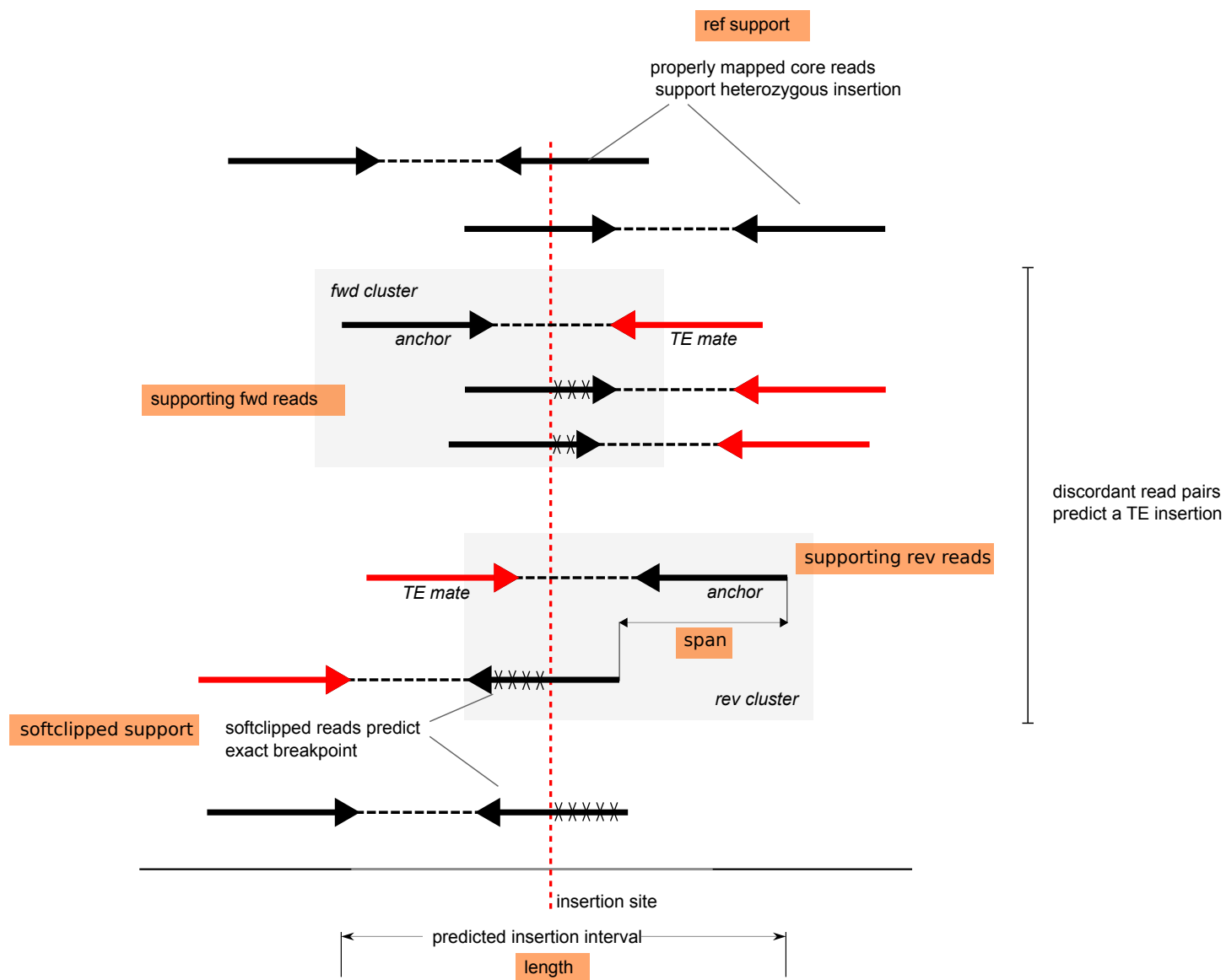

Supplement: Additional file 2: Figure S2. — A) Details of Jitterbug’s prediction method with the metrics used as filtering criteria highlighted in orange. B) True Positive (TP) and False Positive (FP) predictions plotted according to these metrics. For each metric, TP and FP follow different distributions and thresholds can be determined to eliminate FP without excessive loss of TP. (ZIP 55 kb) [file 12864_2015_1975_MOESM2_ESM.zip › add 2/Supp2A.pdf]

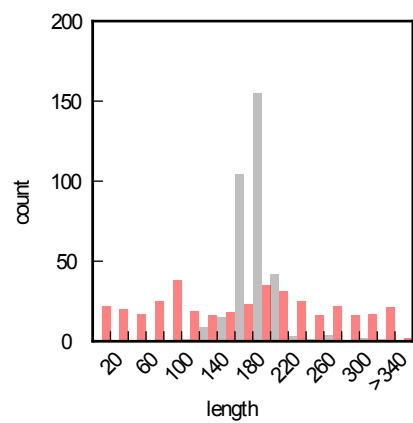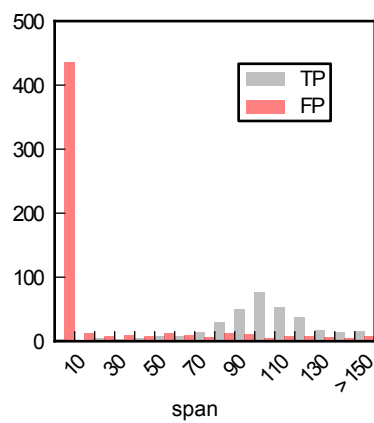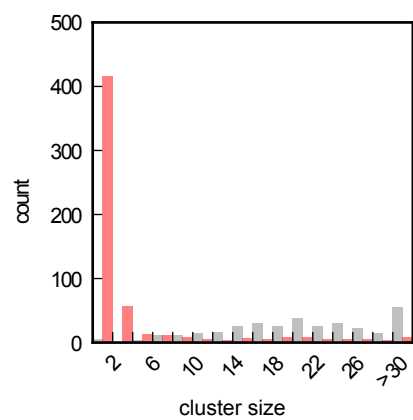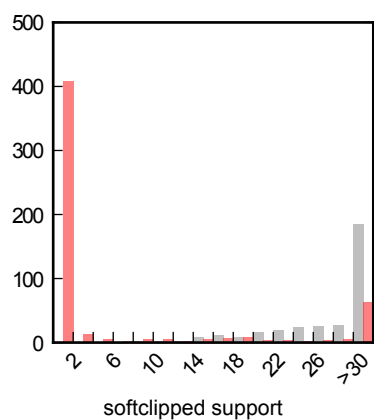

Supplement: Additional file 2: Figure S2. — A) Details of Jitterbug’s prediction method with the metrics used as filtering criteria highlighted in orange. B) True Positive (TP) and False Positive (FP) predictions plotted according to these metrics. For each metric, TP and FP follow different distributions and thresholds can be determined to eliminate FP without excessive loss of TP. (ZIP 55 kb) [file 12864_2015_1975_MOESM2_ESM.zip › add 2/Supp2B.pdf]

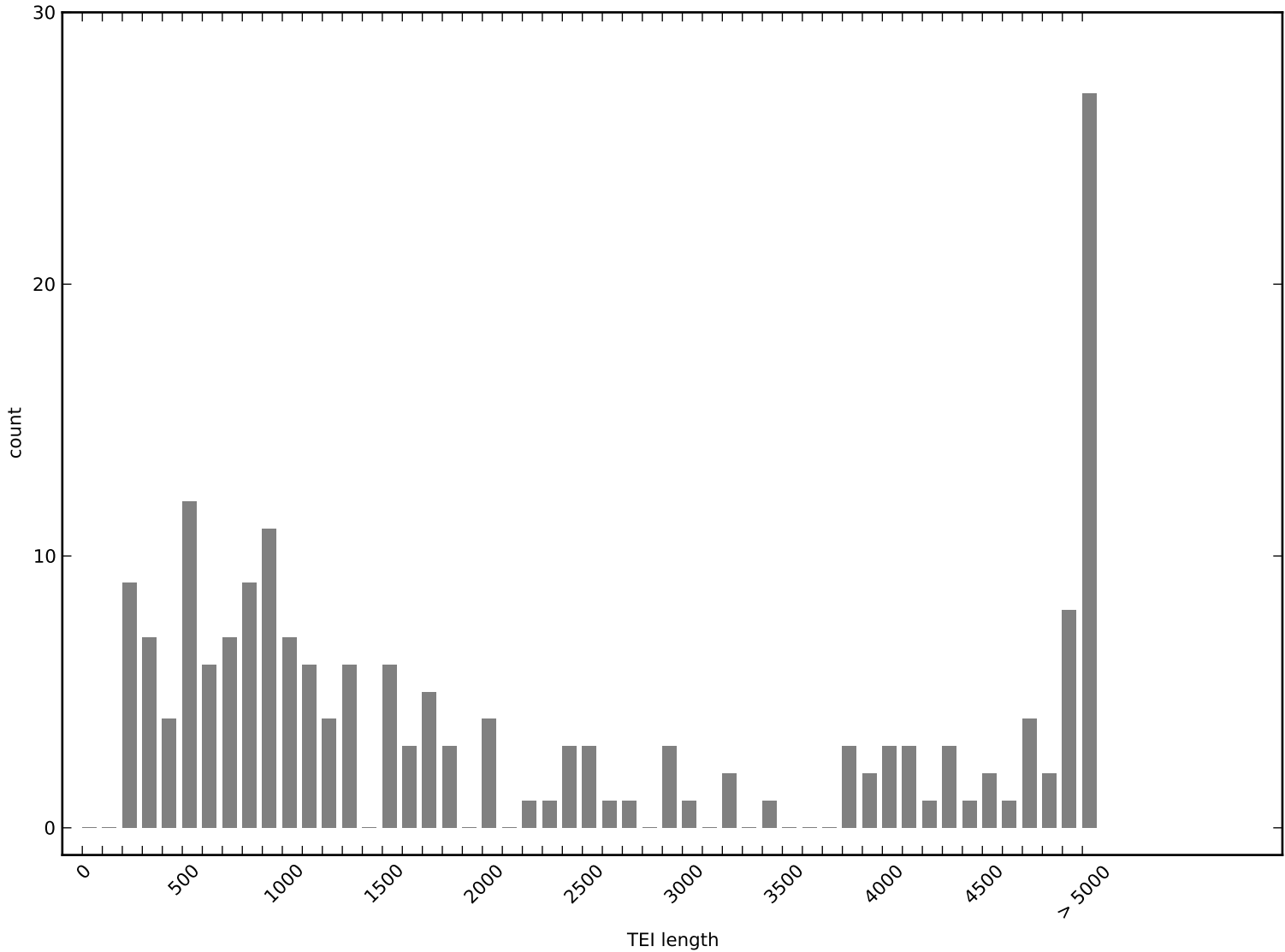

Supplement: Additional file 3: Figure S4. — Distribution of the length of pre-assembled Ler-1 PacBio reads. (PDF 10 kb) [file 12864_2015_1975_MOESM3_ESM.pdf]

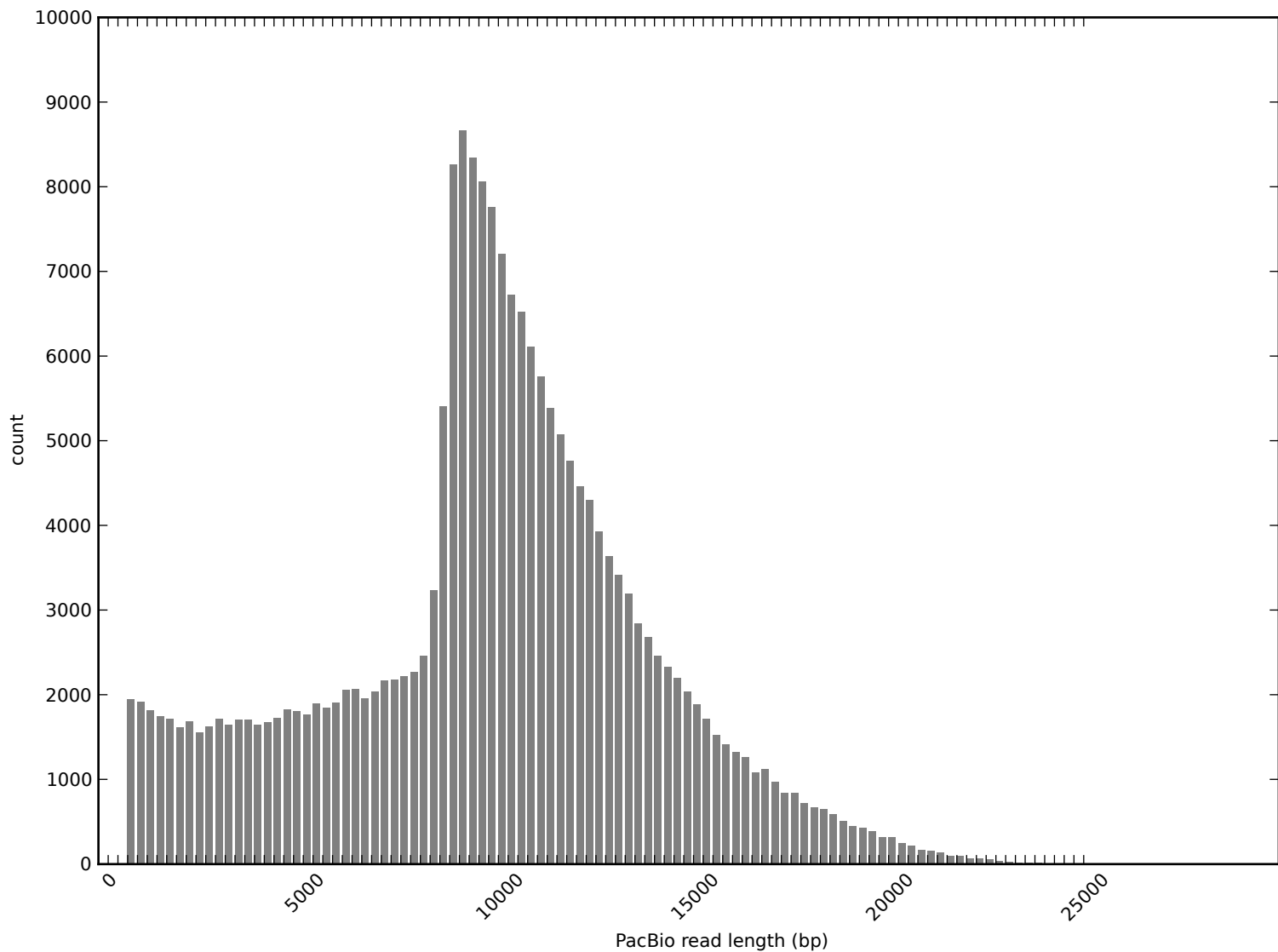

Supplement: Additional file 4: Figure S3. — Distribution of the length of TE insertions identified in Landsberg erecta (Ler-1) compared to Columbia-0, as determined by alignment of predicted TEI loci with Ler-1 PacBio reads. (PDF 48 kb) [file 12864_2015_1975_MOESM4_ESM.pdf]

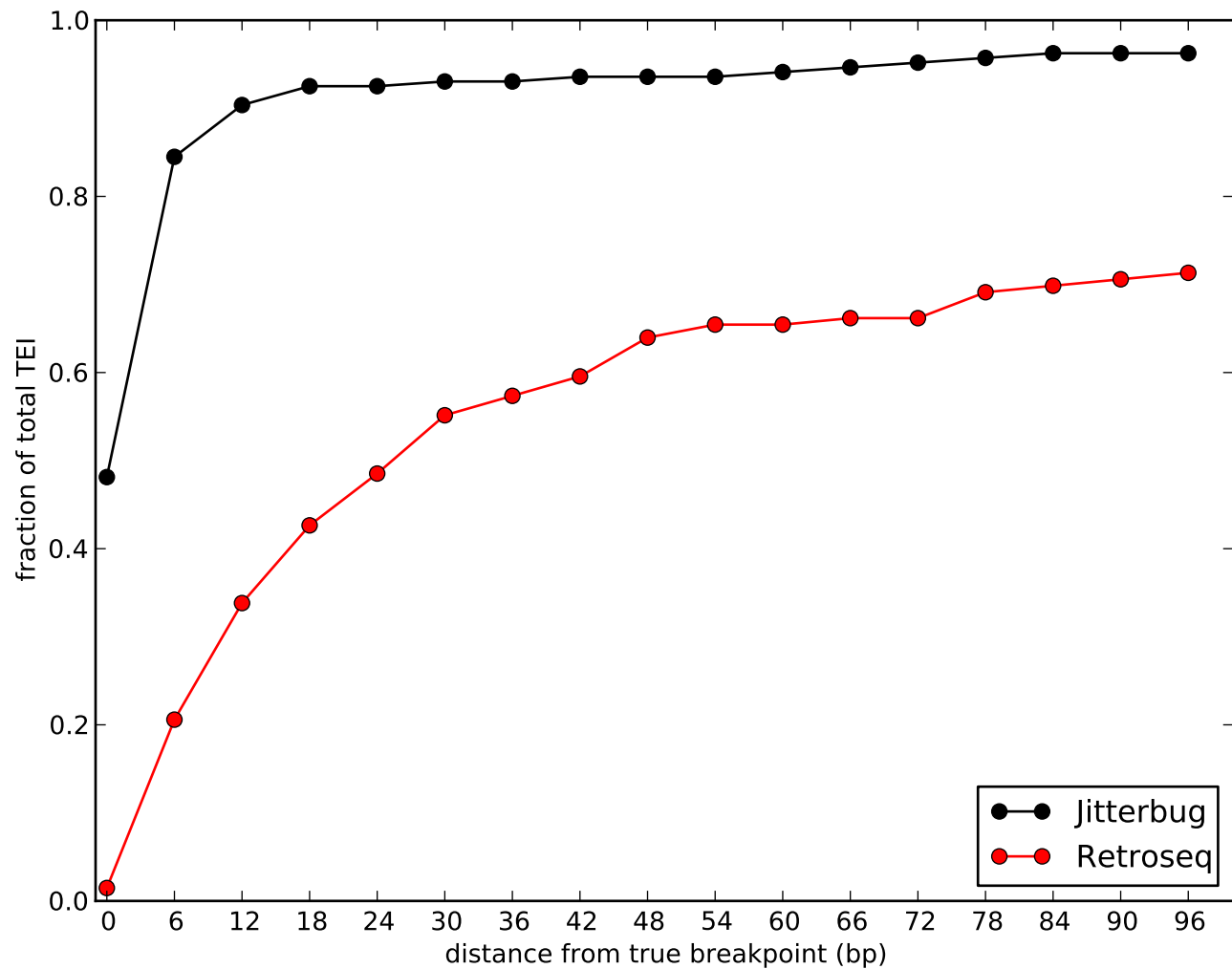

Supplement: Additional file 5: Figure S9. — A) characteristic signature of Illumina short read mapping in the case of a target site duplication generated by the insertion of a mobile element. B) Example of a TEI with TSD in Ler-1 compared to Columbia-0: IGV screenshots and BLAT alignments of Illumina and PacBio sequences, mapped to the reference, respectively. Jitterbug calls the breakpoint as the position with highest softclipped reads support, which can be either side of the TSD, 5’ in the first example and 3’ in the second. BLAT reports the 3’ position on the forward (reference) strand as the breakpoint. The difference in breakpoint position determined by these two methods in many cases corresponds to either zero or the length of the TSD. (PDF 15 kb) [file 12864_2015_1975_MOESM5_ESM.pdf]

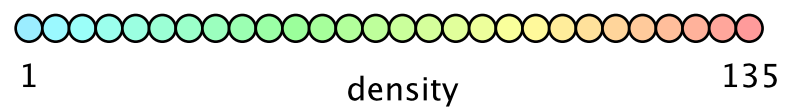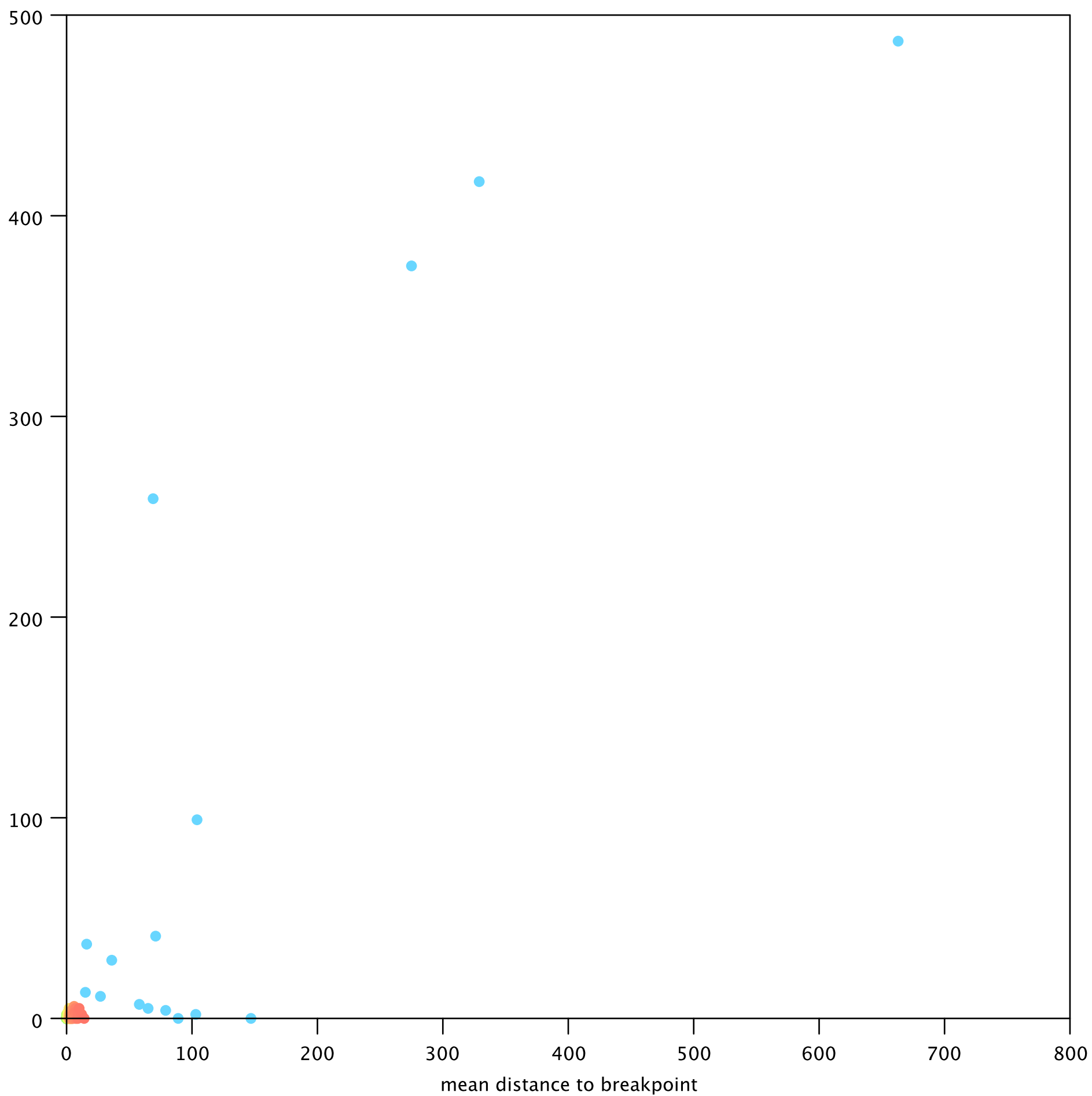

Supplement: Additional file 6: Figure S5. — Fraction of total TEI compared to the distance between the predicted breakpoint and that determined by alignment of the sequences flanking the TEI loci to PacBio reads, for Jitterbug and RetroSeq (ZIP 27 kb) [file 12864_2015_1975_MOESM6_ESM.zip › add 6/Supp6A.pdf]

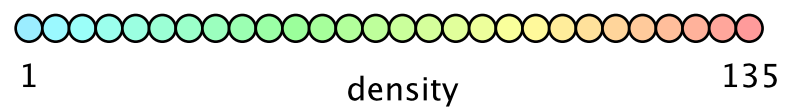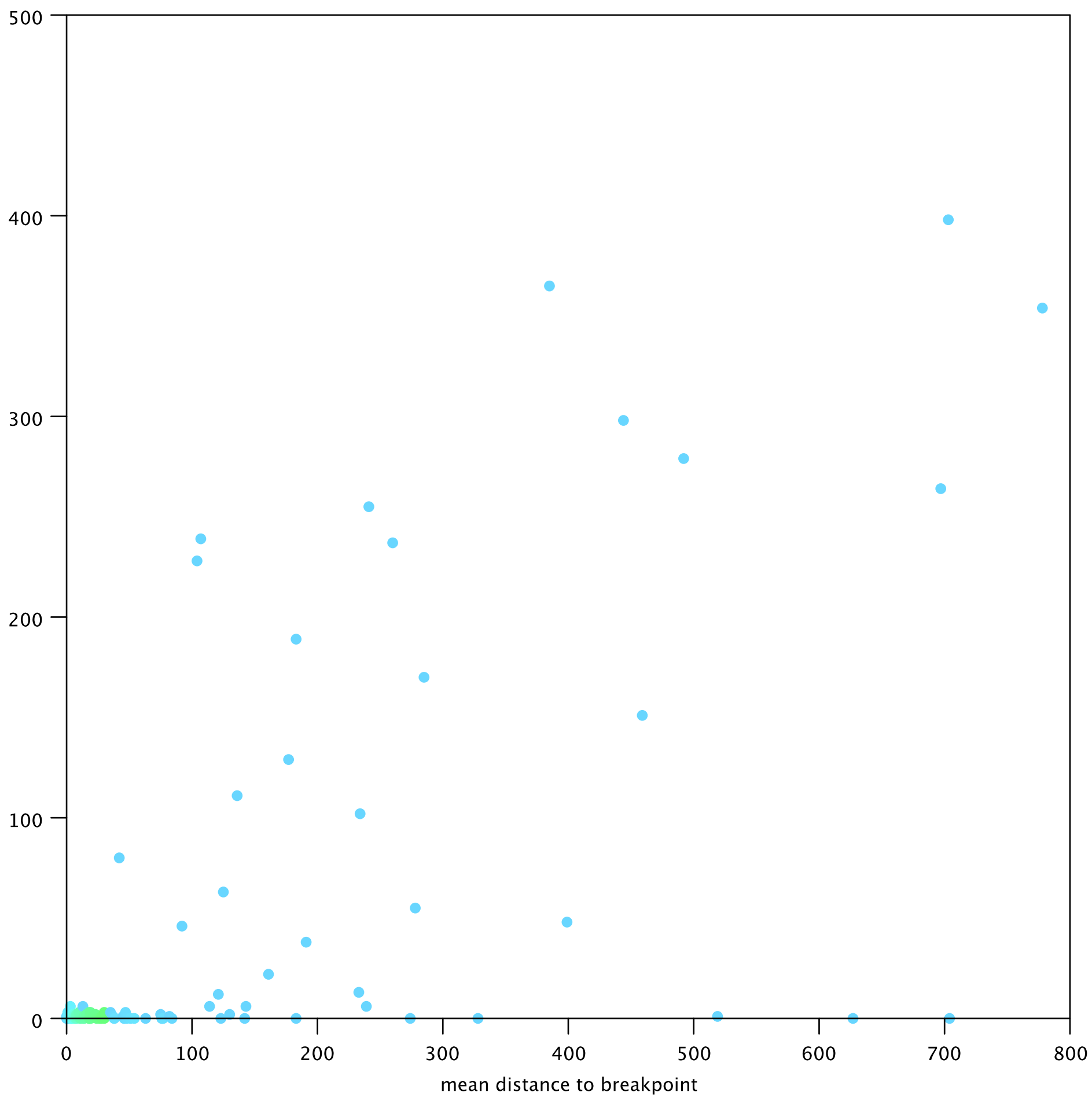

Supplement: Additional file 6: Figure S5. — Fraction of total TEI compared to the distance between the predicted breakpoint and that determined by alignment of the sequences flanking the TEI loci to PacBio reads, for Jitterbug and RetroSeq (ZIP 27 kb) [file 12864_2015_1975_MOESM6_ESM.zip › add 6/Supp6B.pdf]

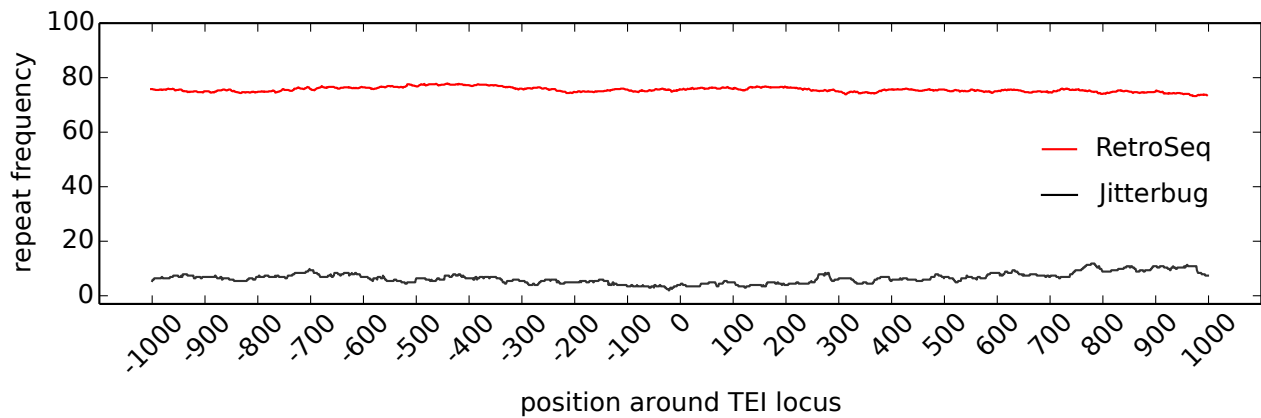

Supplement: Additional file 7: Figure S6. — For TEI which align to > 3 PacBio reads, the standard deviation of the distances between the predicted breakpoint and that determined by the alignment for each read was plotted against the mean. A deviation of 0 indicates that the same breakpoint is predicted in all alignments. A) Jitterbug: the cluster of points around 0, 0 indicate that most alignments are highly concordant between the set of reads and are close to the predicted breakpoint. B) RetroSeq: the spread of points is consistent with the fact that most TEI are predicted in annotatated TEs, which by their repetitive structure would allow multiple possible alignments. (PDF 19 kb) [file 12864_2015_1975_MOESM7_ESM.pdf]
