# Supplementary material for: Jitterbug: somatic and germline transposon insertion detection at single-nucleotide resolution
Source: BMC Genomics. 2015 Oct 12;16:768. doi: 10.1186/s12864-015-1975-5 (PMC4603299; doi:10.1186/s12864-015-1975-5)
Supplement: Additional file 8: Figure S7. — Percent of TEI flanking sequences masked by RepeatMasker. On average, 80 % of the sequences flanking RetroSeq TEI (red) are masked, compared to 10 % for Jitterbug (black). (PDF 19 kb) [file 12864_2015_1975_MOESM8_ESM.pdf]

| | runtime (h:m:s) | |
|---|---|---|
| | 8 CPU, 16G RAM | |
| | Jitterbug | RetroSeq |
| 10X | 0:01:58 | 0:22:59 |
| 20X | 0:03:46 | 1:15:06 |
| 40X | 0:06:52 | 3:41:07 |
