# Supplementary material for: Jitterbug: somatic and germline transposon insertion detection at single-nucleotide resolution
Source: BMC Genomics. 2015 Oct 12;16:768. doi: 10.1186/s12864-015-1975-5 (PMC4603299; doi:10.1186/s12864-015-1975-5)
Supplement: Additional file 9: Figure S8. — Runtime benchmark (hour:min:sec) of Jitterbug and RetroSeq on the simulated ND sample in hg19 (limited to chromosomes 1 and 2), at various coverage depths. (PDF 138 kb) [file 12864_2015_1975_MOESM9_ESM.pdf]

A

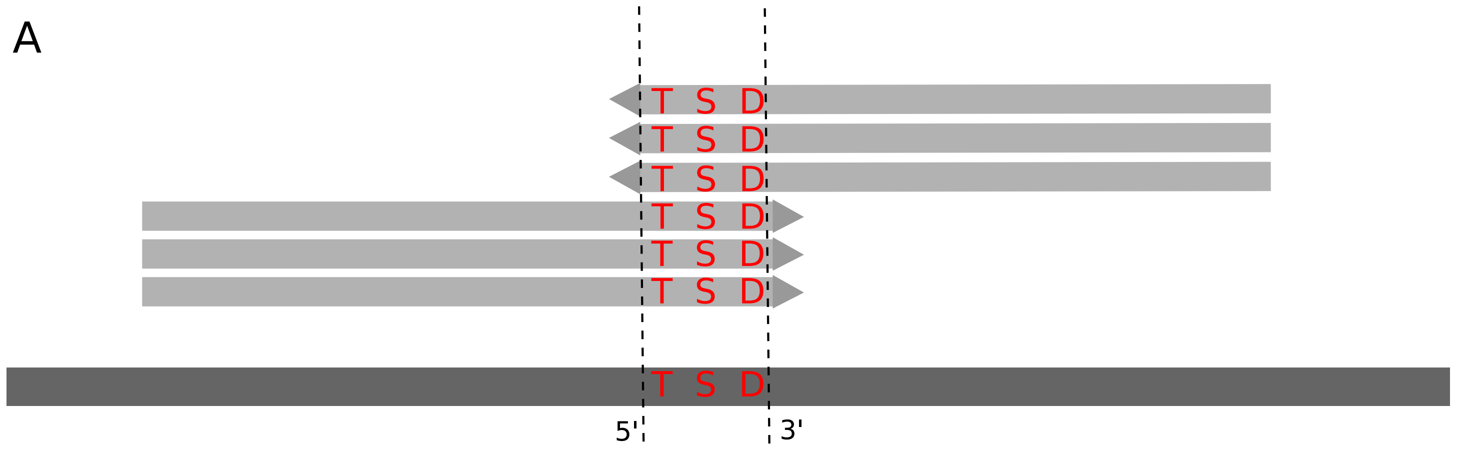

B

Example 1: 5bp distance reported between Jitterbug and PacBio alignment breakpoints

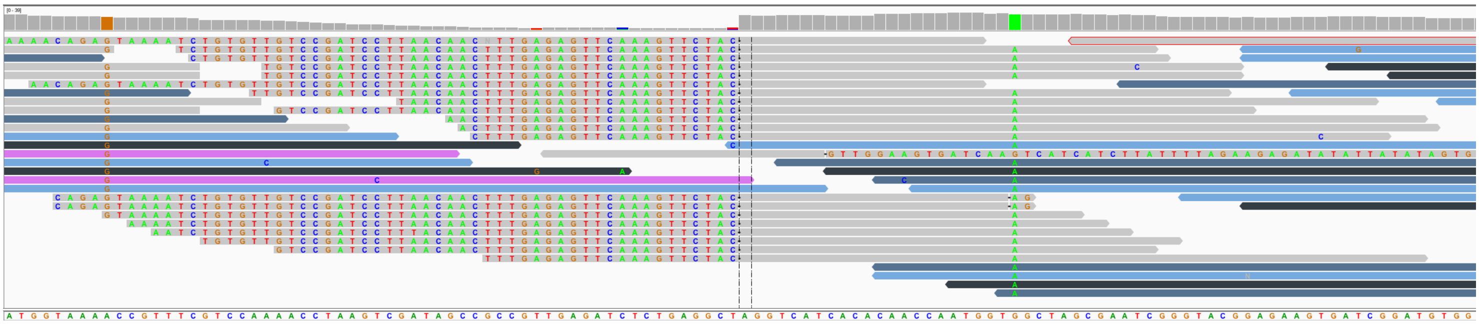

IGV screenshot: Ler-1 Illumina reads aligned to Col0 reference

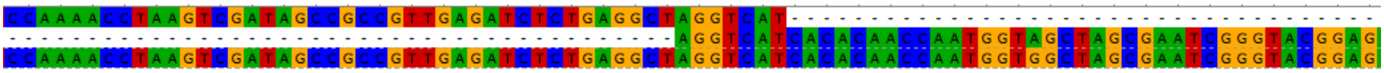

Split BLAT alignment of one Ler-1 PacBio read (top two sequences) to Col0 reference (bottom sequence)

Example 2: 0bp distance reported between Jitterbug and PacBio alignment breakpoints

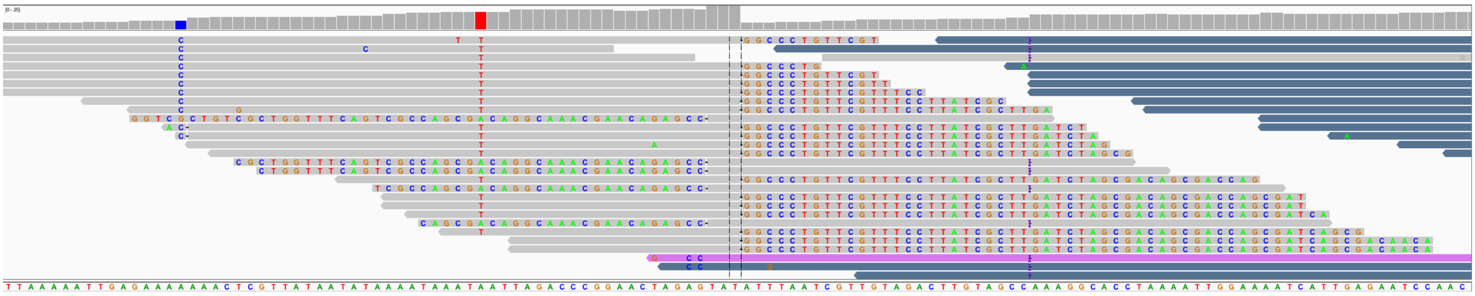

IGV screenshot: Ler-1 Illumina reads aligned to Col0 reference

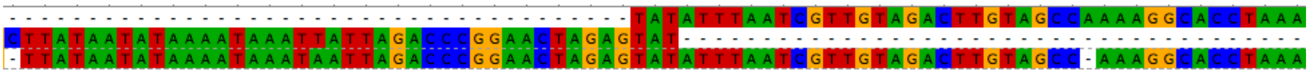

Split BLAT alignment of one Ler-1 PacBio read (top two sequences) to Col0 reference (bottom sequence)
